# TMO-Net: an explainable pretrained multi-omics model for multi-task learning in oncology

**DOI:** 10.1186/s13059-024-03293-9

**Published:** 2024-06-06

**Authors:** Feng-ao Wang, Zhenfeng Zhuang, Feng Gao, Ruikun He, Shaoting Zhang, Liansheng Wang, Junwei Liu, Yixue Li

**Affiliations:** 1 Key Laboratory of Systems Health Science of Zhejiang Province, School of Life Science, Hangzhou Institute for Advanced Study, University of Chinese Academy of Sciences, Hangzhou, 310024 China; 2 Guangzhou National Laboratory, Guangzhou, 510005 China; 3 Department of Computer Science at the School of Informatics, Xiamen University, Xiamen, 361005 China; 4 Department of Colorectal Surgery, The Sixth Affiliated Hospital, Sun Yat-Sen University, Guangzhou, 510655 China; 5 Shanghai Artificial Intelligence Laboratory, Shanghai, 200433 China; 6 Biomedical Innovation Center, The Sixth Affiliated Hospital, Sun Yat-Sen University, Guangzhou, 510655 China; 7 BYHEALTH Institute of Nutrition & Health, Guangzhou, 510000 China; 8 Shanghai Institute of Nutrition and Health, Chinese Academy of Sciences, Shanghai, 200030 China; 9 GZMU-GIBH Joint School of Life Sciences, The Guangdong-Hong Kong-Macau Joint Laboratory for Cell Fate Regulation and Diseases, Guangzhou Medical University, Guangzhou, 511436 China; 10 School of Life Sciences and Biotechnology, Shanghai Jiao Tong University, Shanghai, 200240 China; 11 Collaborative Innovation Center for Genetics and Development, Fudan University, Shanghai, 200433 China; 12 Shanghai Institute for Biomedical and Pharmaceutical Technologies, Shanghai, 200032 China

**Keywords:** Multi-omics, Model pre-training, Transfer learning, Prognosis prediction, Cancers

## Abstract

Cancer is a complex disease composing systemic alterations in multiple scales. In this study, we develop the Tumor Multi-Omics pre-trained Network (TMO-Net) that integrates multi-omics pan-cancer datasets for model pre-training, facilitating cross-omics interactions and enabling joint representation learning and incomplete omics inference. This model enhances multi-omics sample representation and empowers various downstream oncology tasks with incomplete multi-omics datasets. By employing interpretable learning, we characterize the contributions of distinct omics features to clinical outcomes. The TMO-Net model serves as a versatile framework for cross-modal multi-omics learning in oncology, paving the way for tumor omics-specific foundation models.

**Supplementary Information:**

The online version contains supplementary material available at 10.1186/s13059-024-03293-9.

## Background

Cancer is predominantly initiated with the aberrant regulation of tumor suppressor genes or proto-oncogenes, leading to systemic cellular alterations and eventual tumor cell proliferation and metastasis [1]. Recent advancements in diagnostic and examination methods enable the profiling of local tumor tissues using multiple modalities, including genomics, epigenomics, transcriptomics, proteomics, and more [2]. All these datasets provided abundant information for comprehending the pathogenesis and progression of cancers, posing a challenge in their integrated analysis for precision oncology studies [2, 3]. Furthermore, the progress in high-throughput sequencing technologies and the establishment of large-scale cancer research platforms, including The Cancer Genome Atlas (TCGA) [4] and the International Cancer Genome Consortium (ICGC) [5] projections, have amassed a wealth of paired multi-omics cancer datasets spanning various cancer types. Integrating the clinical phenotypes of individual patient cohorts allows us to delve deeper into the underlying genomic regulations, identifying crucial molecular features that are closely associated with tumorigenesis and disease progression [6].

Multi-omics data learning is another major challenge for effectively utilizing these multi-omics datasets available for specific cancer cohorts [7]. Various methods have been developed to integrate these high-dimensional datasets for predicting specific clinical outcomes [8–11]. However, a significant limitation of most existing methods is their reliance on study-related patient cohort datasets, which often have smaller cohort sizes compared to the feature counts of these high-dimensional data modalities, leading to the potential issue of the “curse of dimensionality” [12]. Besides, the obtained data modalities can be varied across different cancer studies, adding extra complexity to data integration and analysis, and hindering data integration across multi-omics datasets. Cross-omics data inference presents a valuable strategy for integrating and understanding multi-omics datasets [13–17]. Through the imputation of missing omics data within individual samples, we can achieve more comprehensive sample representations, thereby advancing various downstream prediction tasks.

While pre-training frameworks have demonstrated success in enhancing the performance of deep learning models in specific tasks, including prognosis prediction [18], cancer dependency gene prediction [19], and others, the transferability of these models to diverse downstream tasks remains impractical. In addition, the development of foundation models in the biomedical field has significantly expanded the applications of deep learning methods in various domains, including single-cell omics [20], pathology image analysis [21], retinal image analysis [22], medical reports [18], and so on. The capability of foundation models to pre-train with large-scale datasets and fine-tune with task-specific labeled datasets allows for the learning of fundamental information within specific data modalities while maintaining a high model general applicability [18]. Therefore, there is a pressing need for a universal deep learning framework capable of seamlessly integrating diverse and complex multi-omics pan-cancer datasets. Such a framework should be designed to learn the underlying gene regulatory mechanisms across different modalities through model pretraining, ensuring broad applicability across a range of tasks.

Here, we introduce TMO-Net, an explainable, pre-trained deep learning model specifically designed for the integration of multi-omics cancer datasets and further adapting into multiple oncology downstream tasks. Pretrained initially with multi-omics pan-cancer datasets, the TMO-Net model can learn the underly genomic regulations between molecular features across gene mutation, mRNA expression, copy number variation (CNV), and DNA methylation. Notably, this model incorporated a cross-omics fusion network, which adeptly learns the connections between latent variables from different data modalities, enables missing modality data inference, and expands its broad applications in oncology research. With different labeled multi-omics cancer datasets, we further adapted and fine-tuned the TMO-Net model into different oncology downstream tasks, including cancer subtype classification, metastasis prediction, drug response prediction, and prognosis prediction. Our experiments reveal that the pre-trained TMO-Net model outperforms most state-of-the-art models and also the TMO-Net model trained from scratch. Moreover, the pre-trained TMO-Net model demonstrates better learning of tumor representations across pan-cancer datasets, showcasing its ability to extract joint representations from diverse multi-omics datasets. Leveraging explainable deep learning methods, we characterized the impacts of different molecular features on various clinical outcomes for cancer patients. The TMO-Net model highlighted large-scale data pretraining as a crucial method for the future of multi-omics cancer research and contributed to the development of generalist multi-omics cancer foundation models.

## Results

### Model framework

**Fig. 1:**
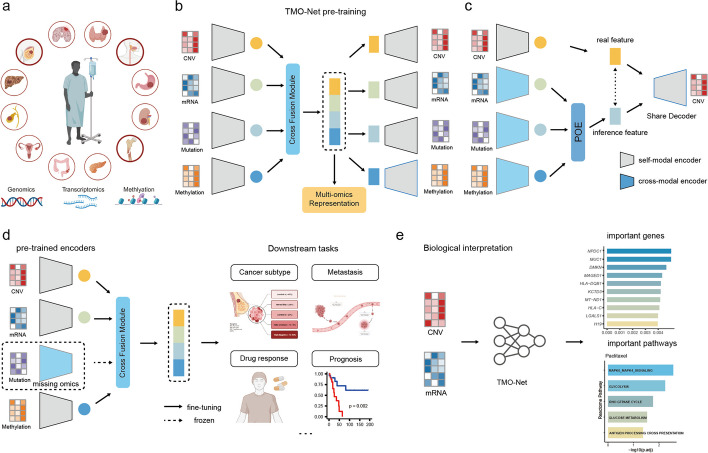
Overview of the TMO-Net model. **a** Pan-cancer multi-omics dataset utilized in the pre-training stage, comprising gene mutation, mRNA expression, copy number variation (CNV), and DNA methylation modalities. **b** The architecture of the TMO-Net model, encompasses self-modal variational autoencoders, cross-modal variational autoencoders, and the “Cross Fusion Module”. **c** Schematic of the Cross Fusion Module for cross-modal learning and fusion, devised for aligning latent embedding from different modalities, cross-modal learning to infer the missing modalities, and fusing the complete multi-omics embedding. **d** Diagram of utilizing pre-trained TMO-Net model for fine-tuning in multiple downstream tasks through transfer learning, accommodating missing modalities. **e** The biological interpretations analysis for the multi-omics features via analyzing the global gene attributions in integrated gradients across datasets and utilizing the importance scores of individual genes to characterize pathway enrichments

To establish a comprehensive analytical framework for extracting clinical information from multi-omics cancer datasets, we developed the Tumor Multi-Omics pre-trained Network (TMO-Net) model. This model was specifically designed to be pre-trained with large-scale pan-cancer multi-omics datasets and learn the relationships among individual omics features. Meanwhile, the model was engineered to accommodate the learning of incomplete omics data, making it suitable for a broad spectrum of cancer-related deep learning tasks (Fig. 1a, b, and see the “Methods” section). This model utilized multiple variational autoencoders (VAEs) for capturing associations within self-modal and cross-modal features [23]. Additionally, a “Cross Fusion Module” was integrated to efficiently align latent spaces from different modalities and facilitate the inference of missing modalities [24] (Fig. 1c). These encoders collaboratively produced joint embeddings for each omics type, which were then fused to generate comprehensive joint multi-omics sample embeddings. The detailed description of the TMO-Net model framework was introduced in the “TMO-Net framework” section in the “Methods” section.

The TMO-Net model was pre-trained using processed large-scale pan-cancer multi-omics datasets [19], which encompass paired data from 32 cancer types, involving 8174 samples, including gene mutation, mRNA expression, copy number variation (CNV), and DNA methylation modalities. The model underwent pre-training using a multi-objective, self-supervised learning framework. The comprehensive loss function comprises several components: (1) *Self-ELBO loss*: this loss aims to learn the latent variance distribution while enabling accurate reconstruction of the input data. (2) *Cross-modal ELBO Loss*: this component focuses on capturing the associations between different omics layers and enables the model to reconstruct specific modalities from others. (3) *Discriminator Loss*: this loss compels the model to discriminate between original and reconstructed data and align latent representations across modalities. (4) *Contrastive Loss*: this loss aligns embeddings of the samples with the same tumor subtype while differentiating them from others. Throughout the pre-training phase, we refined the hyperparameters of these distinct model losses to enhance the capture of patient-level representations in cancer datasets. Detailed descriptions of the model losses, hyper-parameters, and pretraining strategies can be found in the “Model pre-training loss” and “TMO-Net pre-training process” sections of the “Methods” section. Furthermore, the joint embeddings learned by TMO-Net, incorporating diverse multi-omics datasets, along with the multimodal encoders, were subsequently employed to adapt to various downstream cancer tasks and models, especially for independent and limited-size cancer patient cohorts (Fig. 1d). Additionally, we integrated model interpretation methods to uncover the significance of specific omics molecular features with the predicted outcomes, aiding in the identification of cancer-related multi-omics mechanisms (Fig. 1e).

### TMO-Net model enables improved cancer representation learning

**Fig. 2:**
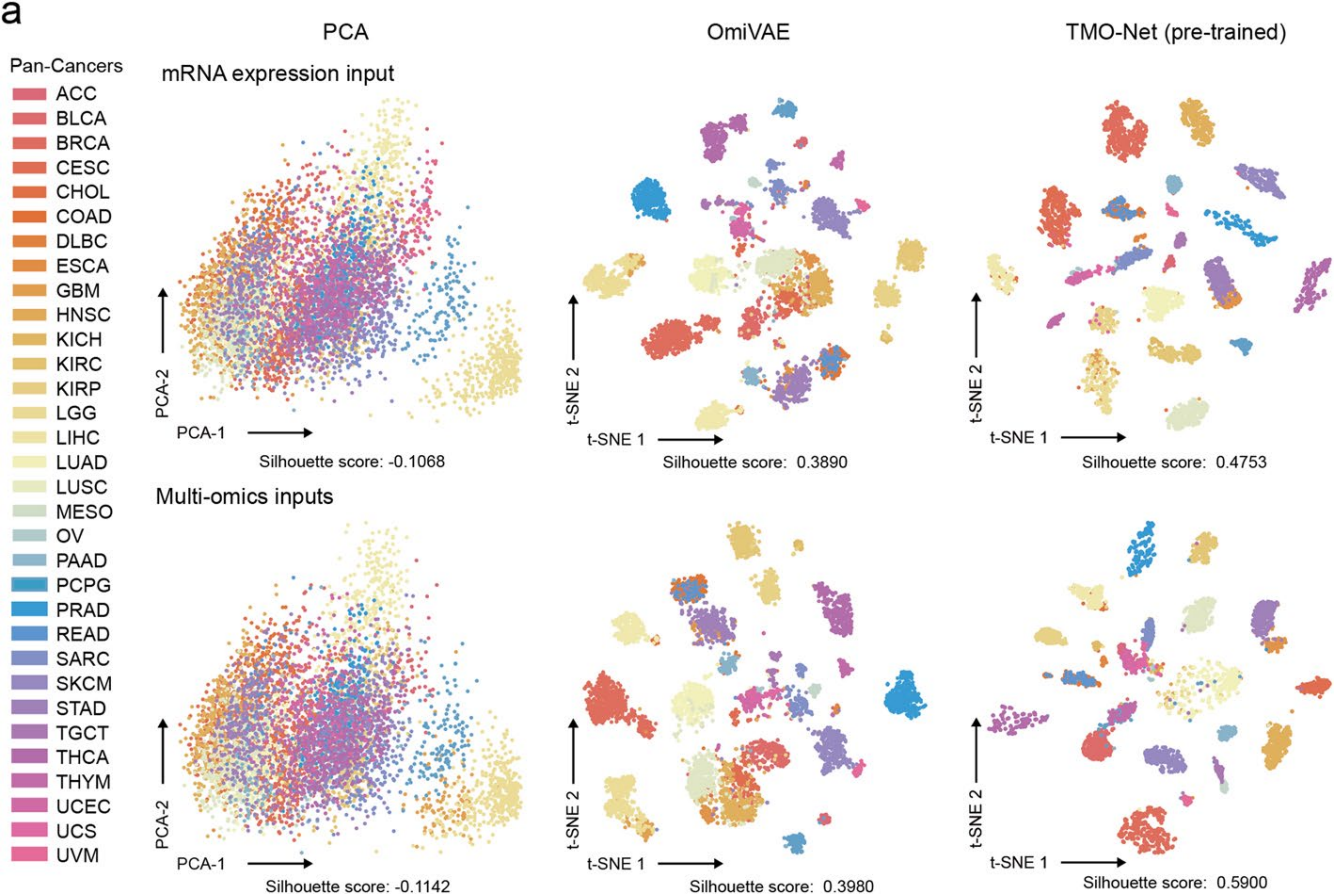
Comparison of pan-cancer sample representation learning. t-SNE plots of pan-cancer omics representation learning with TMO-Net (pre-trained) and baseline methods, colored by cancer type. The upper plots illustrate representations based solely on mRNA expression input and the lower plots with multi-omics inputs. Silhouette scores were applied to compare the model performance in representation learning

We first assessed the performance of the TMO-Net model in learning representations of multi-omics or incomplete-omics cancer datasets. The heterogeneous pathogenesis and phenotypes of different cancers can result in the distinct molecular profiles of tumor samples; we then utilized the learned joint embedding of pan-cancer multi-omics datasets of various methods to compare their capabilities in segregating samples with distinct cancer subtypes via unsupervised learning (Fig. 2a). The silhouette score was utilized to evaluate the effectiveness of each method in segregating samples with distinct cancer subtypes [25]. Our results demonstrate that the latent embeddings inferred by the TMO-Net model can better represent samples from distinct cancers with the highest silhouette score in both gene expression and multi-omics datasets, in contrast to that with PCA and OmiVAE [26] (Fig. 2a).

To further characterize the representation learning capabilities of various methods, we undertook a downstream classification task to predict cancer subtypes using different pre-training datasets. After model pre-training, the encoders of the OmiVAE and TMO-Net models were frozen, along with the pre-calculated principal components, we then compared the classification performance of the learned embeddings via an XGBoost [27]. Notably, the TMO-Net model outperformed the OmiVAE model across multi-omics and gene expression datasets, achieving the highest average F1 score of 0.751 (Additional file 2: Table S1). Moreover, to investigate the impact of pre-training data scales on modal representation learning, we utilized a classification network to assess the performance of learned sample embeddings by TMO-Net models pre-trained with subset training multi-omics datasets, ranging from 10 to 100%. Our findings indicated a strong correlation between the data scale and the representation learning capability of the TMO-Net model in classifying cancer subtypes within validating datasets for different pre-training datasets (Additional file 1: Fig. S1, Additional file 2: Table S2). Besides, we applied the Integrated Gradients [28] algorithm to evaluate the contribution of various omics in predicting cancer subtypes. By aggregating the IG values of all features across different omics to cancer-specific prediction neurons, we revealed that gene expression and gene methylation omics contributed significantly more to cancer classification compared to gene mutation and CNV omics (Additional file 2: Tables S3).

### TMO-Net model enables cross-modal inference

**Fig. 3:**
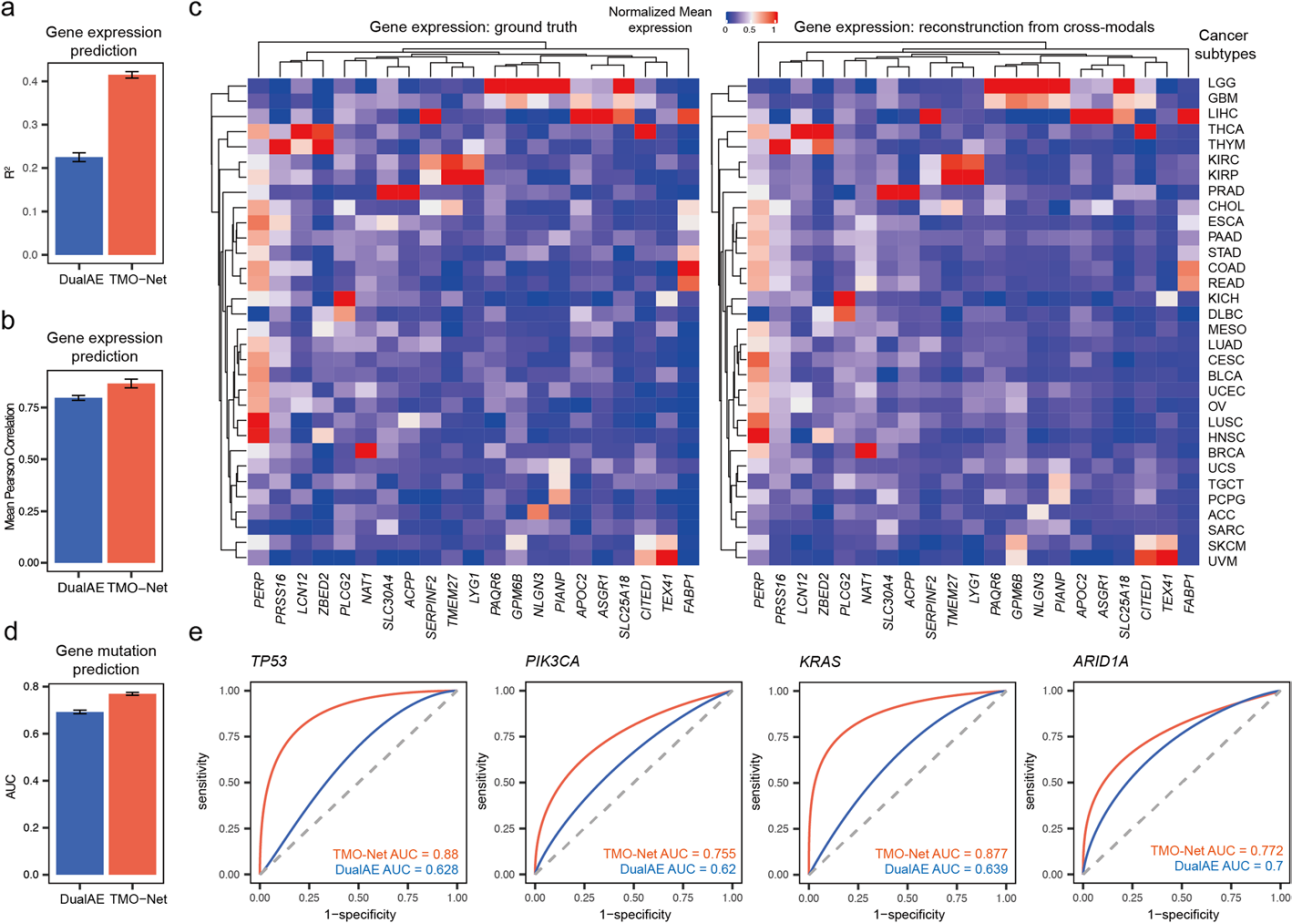
The ability of the TMO-Net model in cross-omics generation. **a**, **b** Comparison of the averaged coefficient of determination (*R*^2^) (**a**) and Pearson’s correlation coefficients (**b**) of raw and reconstructed gene expression values from other modalities. **c** Heatmap plot of the raw and reconstructed gene expressions in selected genes across cancer subtypes. **d** Comparison of the averaged AUC score of predicted gene mutations in the top 50 most mutated genes. **e** Receiver operating characteristic (ROC) plots of the prediction result of typical cancer mutations. Fivefold cross-validation results were presented in **a**, **b**, and **d**, with mean ± SD

The cross-modal learning module of the TMO-Net model facilitates interaction learning among different modalities through cross-modal data regeneration. We then assessed the TMO-Net model’s ability to reconstruct gene expressions and mutations from alternative modalities. In comparison to a baseline dual-autoencoder model [29], our results demonstrated that TMO-Net achieves higher coefficients of determination (*R*^*2*^) (Fig. 3a) and Pearson’s correlation coefficients (Fig. 3b) based on the reconstructed gene expression values. Furthermore, representative raw and reconstructed gene expression heatmaps of selected genes across various cancer subtypes further illustrate the gene expression inference capabilities of TMO-Net (Fig. 3c).

We then assessed the performance of the TMO-Net model in predicting gene mutations from other modalities. We subset the top 50 most mutated genes as prediction targets and utilized binary prediction outcomes to denote the gene mutation status of tested samples. The area under the curve (AUC) score was employed to quantify the model’s performance in mutation prediction. Compared to the baseline model, the TMO-Net model achieves superior prediction accuracy (Fig. 3d), particularly in typical cancer-specific gene mutations (Fig. 3e).

### TMO-Net enables breast cancer subtype prediction with model pretraining

**Fig. 4:**
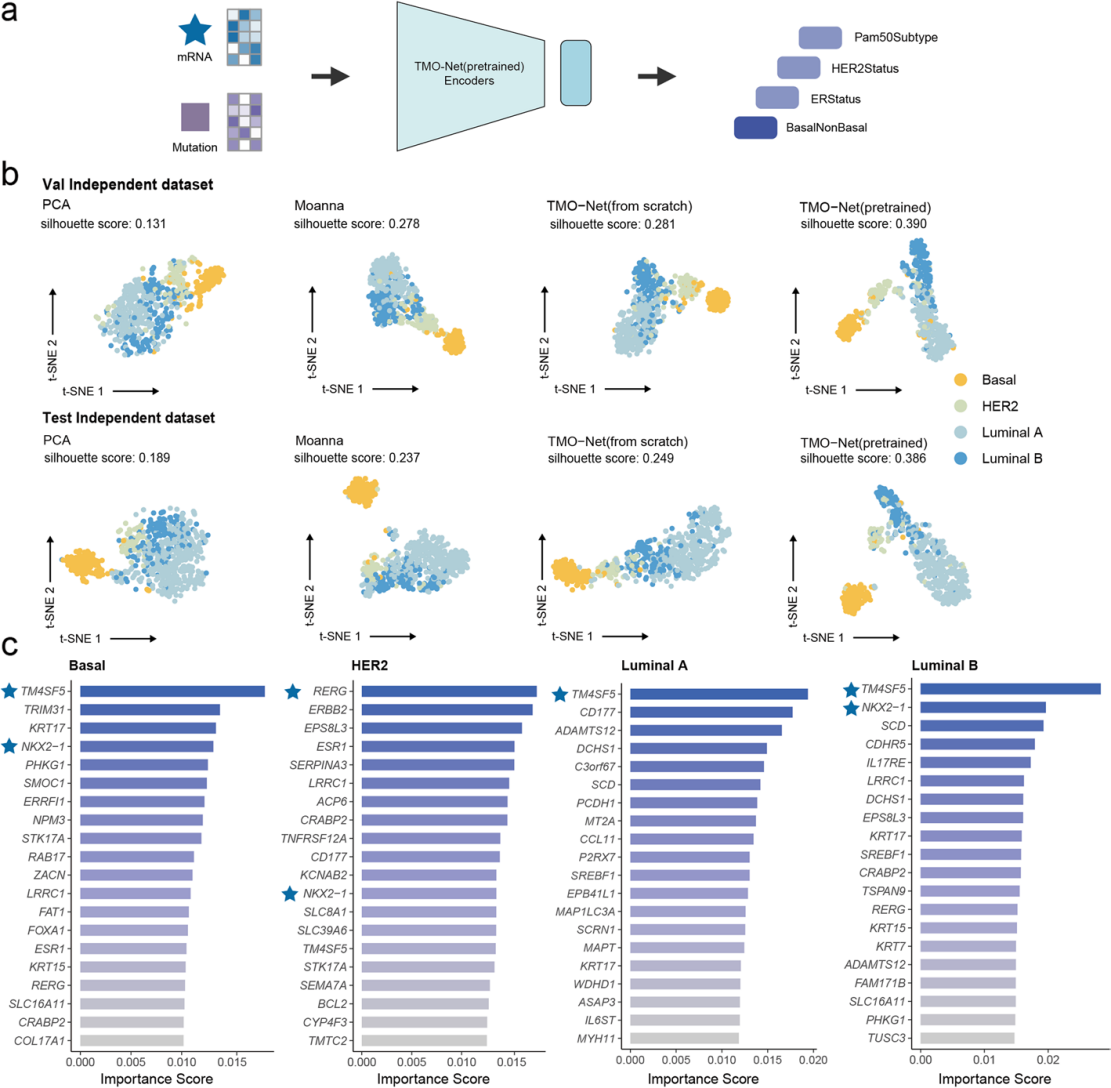
TMO-Net model can enhance breast cancer subtype prediction. **a** Diagram of TMO-Net model adapted for predicting breast cancer subtypes with mRNA and mutation inputs in METABRIC dataset. A Multi-Layer Perceptron (MLP) network was employed to predict the subtype labels, including Basal-NonBasal, ER-Status, HER2-Status, and PAM50Subtype. **b** t-SNE plots of multi-omics representations generated by TMO-Net (pre-trained) and baseline methods, colored by PAM50 subtypes. Silhouette scores were applied to compare the model performance in representation learning. **c** Top-20 most important gene expression features in classifying different PAM50 subtypes of breast cancers. The symbol “star” highlights the crucial genes linked to PAM50 subtypes with literature evidence

To assess the effectiveness and robustness of the TMO-Net model across diverse tumor multi-omics downstream tasks, we employed it to predict the molecular subtypes of breast cancers, which is crucial in devising personalized treatment plans and predicting patient outcomes. We then obtained training and validating datasets from labeled TCGA-BRCA dataset for model fine-tuning and tested with an independent METABRIC dataset [30]. These datasets included cancer samples with identified Basal-like, HER2-enriched, Luminal A, and Luminal B subtype labels, and all samples encompassing gene expression, CNV, and gene mutation modalities. For a comprehensive evaluation of the classification performance of various models, we conducted multiple classification tasks, including ER status, HER2 status, PAM50 subtypes, and Basal status prediction [31] (Fig. 4a). We first evaluated the representation learning capabilities of different models in both the test and validation datasets after fine-tunning with PAM50 subtype prediction task. In comparison to PCA, Moanna [31], and the TMO-Net model without data pre-training, the TMO-Net (pre-trained) model demonstrates the highest silhouette scores in both cancer sample projection analyses with t-SNE [32], highlighting its superiority in latent embedding learning (Fig. 4b). The benchmark results of the classification tasks also revealed that the TMO-Net (pre-trained) model outperforms other baseline methods in most downstream prediction tasks, achieving an average F1-score of 0.921 (Table 1), which supports the notion that pretraining the model with a cancer multi-omics dataset enhances its performance and robustness in cancer-related downstream tasks. Additionally, we assessed the classification performance differences between the TMO-Net model pre-trained with or without BRCA multi-omics cancer datasets, and the results indicated model pre-trained with BRCA datasets had better performance in the breast cancer subtype classification task of METRBRIC dataset, suggesting the critical roles of similar pre-training datasets in TMO-Net model (Additional file 2: Table S4).

**Table 1:** F1 scores of different methods in predicting breast cancer subtypes in validation and test datasets

Methods	ER status		HER2 status		PAM50 subtype		Basal status		Average	
	Val	Test	Val	Test	Val	Test	Val	Test	Val	Test
SVM	0.972	0.942	0.929	0.747	0.863	0.874	0.975	0.974	0.934	0.884
Moanna	0.965	0.947	**0.959**	0.844	0.852	0.852	0.984	0.989	0.940	0.908
TMO-Net (from scratch)	0.975	0.950	0.943	0.840	0.884	0.876	0.984	0.983	0.946	0.912
TMO-Net (pre-trained)	**0.985**	**0.955**	0.953	**0.857**	**0.897**	**0.880**	**0.986**	**0.991**	**0.955**	**0.921**

We also characterized the interpretability of our model in discerning the regulatory roles of various omics features in the development of distinct breast cancer subtypes. We utilized the Integrated Gradients [28] algorithm to calculate attribute scores of each omics feature based on individual sample data and evaluate the global feature scores with their averaged magnitudes [33]. A detailed introduction to model interpretation analysis is available in the “Model interpretation analysis” section of the “Methods” section. By identifying the top 20 most important gene expression features in classifying different PAM50 subtypes of breast cancers (Fig. 4c), we have identified several genes with highly important scores across all breast cancer subtypes. Notably, the *TM4SF5* gene emerged as the most important gene in three or four PAM50 cancer subtypes, which is overexpressed in breast tumors [34] and associated with tumor progression [35]. Additionally, we highlighted the *RERG* gene in the HER2 subtypes, functioning as a tumor suppressor gene in breast cancers [36]. We also identified the *NKX2-1* gene in most cancer subtypes, as it plays a significant role in cancer metabolism and affects tumor aggressiveness [37]. Additionally, we found the *ESR1* gene was annotated highly important in ER-negative breast subtypes (Basal and HER2 subtypes) (Fig. 4c). To characterize the regulatory roles of specific genes in our model, we compared the signed IG values of the canonical genes associated with ER-positive and HER2 subtypes (*ESR1*, *ERBB2*) across breast cancer subtypes. The results validated the signs of IG values correlated with gene expression patterns in diverse breast cancer subtypes (Additional file 1: Fig. S2), with their magnitudes serving as indicators of importance across molecular features.

### TMO-Net enhances predicting cancer samples from primary or metastasis

**Fig. 5:**
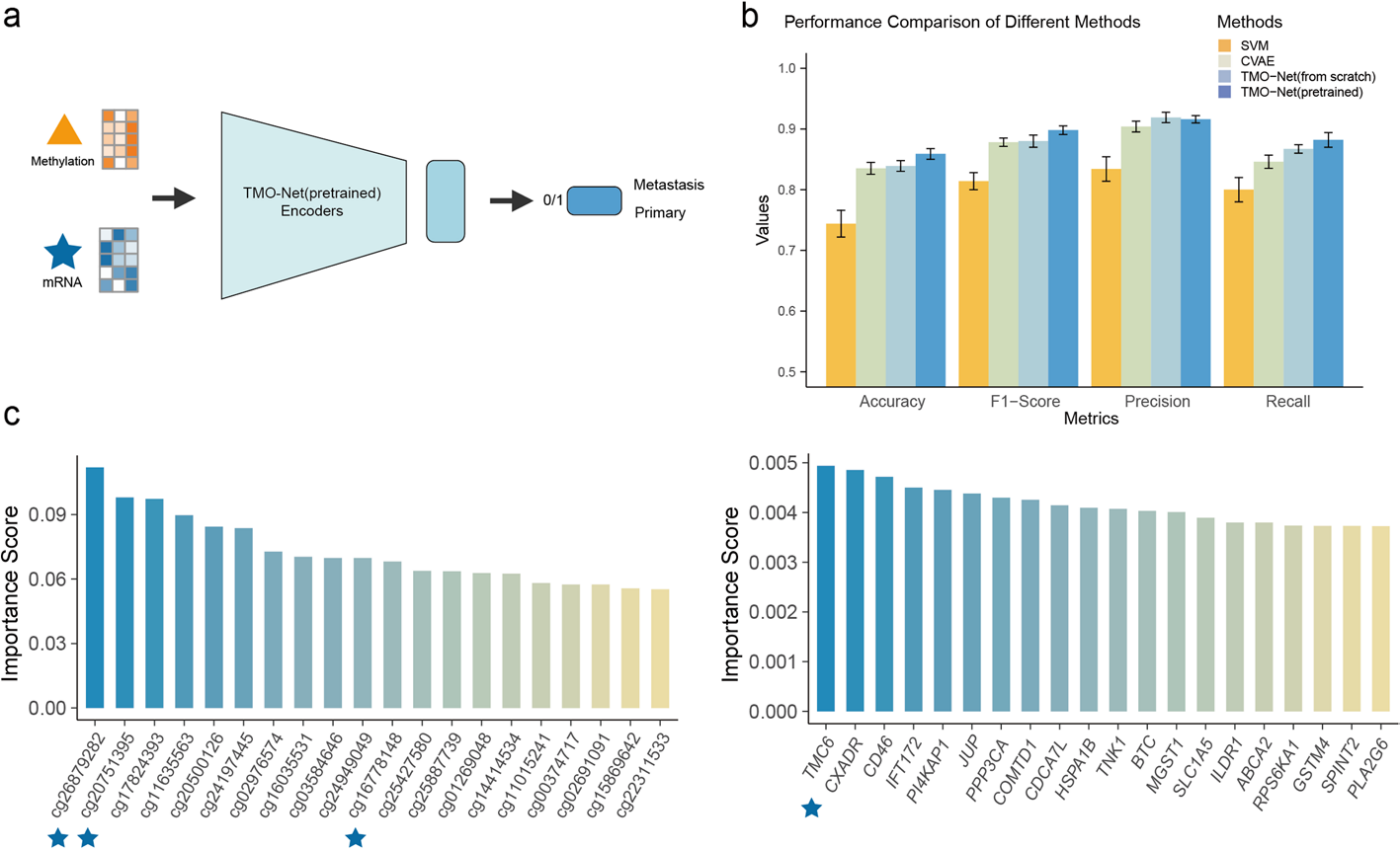
TMO-Net model can separate primary and metastatic cancer samples. **a** Diagram of TMO-Net model adapted for predicting primary or metastatic cancer samples with mRNA and methylation inputs. **b** Performance comparison of different methods in predicting primary or metastatic cancer samples, evaluated by metrics of accuracy, F1-score, precision, and recall. **c** Important molecular features of gene expression and DNA methylation that are associated with tumor metastasis; the symbol “star” denotes the specific gene features and CpG probes linked to the progression and metastasis of various cancers

We then aim to assess the performance of the TMO-Net model in additional cancer downstream tasks and apply it to predict cancer samples from primary tumor sites or distant metastatic organs, a crucial aspect of the treatment and surgery design [38] (Fig. 5a). We obtained balanced samples with annotated distant metastasis or not from the TCGA project, both the gene expression and gene methylation modalities were selected for model training and testing [39].

Various methods, including SVM, CVAE [39], TMO-Net model trained from scratch, and TMO-Net (pre-trained), were employed to segregate metastatic samples from primary ones. Multiple classification performance metrics were utilized to compare the capabilities of these models in predicting metastatic tumor samples (Fig. 5b). Notably, the TMO-Net (pre-trained) model outperforms other models across different metrics, achieving an F1-score of 0.8980, which outperformed SVM, CVAE, and TMO-Net trained from scratch by 0.0831, 0.0191, and 0.0180, respectively (Additional file 2: Table S5). These results underscore the adaptability of the TMO-Net framework to efficiently handle various downstream tasks in multi-omics cancer datasets, showcasing the advantages of model pretraining.

In our analysis of tumor metastasis, we assessed the significance of both gene expression and DNA methylation features (Fig. 5c). We identified the *TMC6* gene as a pivotal factor in tumor metastasis, a role also noted in other metastatic cancers [40]. Moreover, we revealed the feature importance of specific CpG probes: cg26879282, cg20751395, and cg16778148, which were highly associated with tumor metastasis, are located within the genomic regions of the *KCNQ1* gene; the methylation status of *KCNQ1* has been linked to the progression of various cancers [41].

### TMO-Net accurately predicts drug responses

**Fig. 6:**
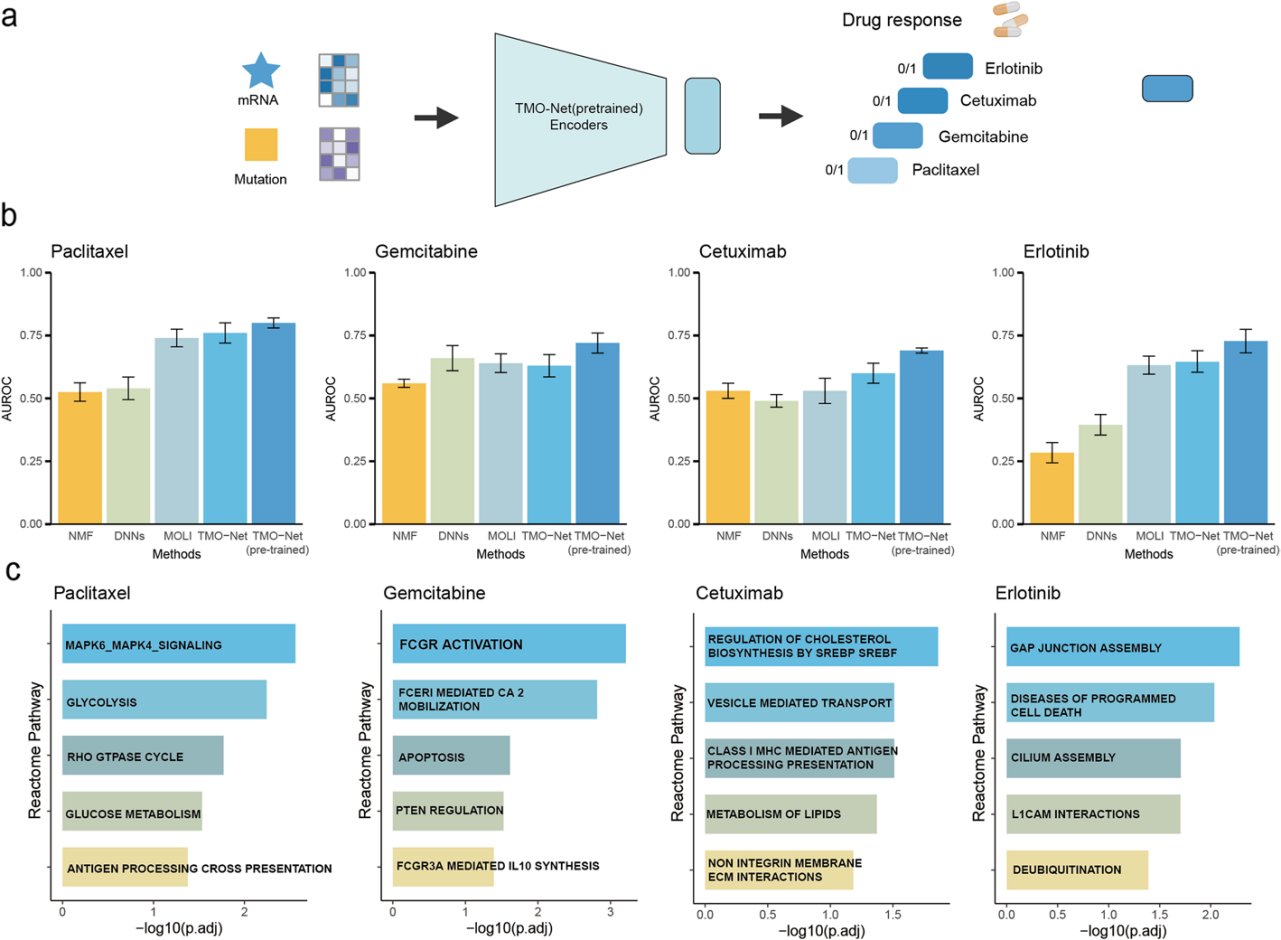
The applications of the TMO-Net model in drug response prediction. **a** Diagram of TMO-Net model adapted for predicting drug responses based on cell-line response datasets with mRNA and mutation inputs. **b** Comparison of the prediction performance of different models in drug response predictions. **c** Bar plots of Reactome pathway enrichments calculated with the importance scores of gene expression features with individual drug responses

We then employed the TMO-Net model to predict drug responses in cancer patients using multi-omics datasets in the Genomics of Drug Sensitivity in Cancer (GDSC) [42] project, to assess the generalization capability of the pre-trained TMO-Net model with only cancer cell line data (Fig. 6a). Binary drug response profiles of gemcitabine, paclitaxel, erlotinib, and cetuximab for the target cell lines in the GDSC dataset were utilized for model training, and an external patient-derived xenograft (PDX) encyclopedia dataset [43] with corresponding drug response labels served as the validation set. We conducted a benchmark analysis of the TMO-Net (pre-trained) model against various baseline methods, including NMF, DNN, MOLI [44], and the TMO-Net model trained from scratch. The ROC-AUC scores of different methods across all drug response tasks revealed that the TMO-Net (pre-trained) model outperforms all other methods, achieving the highest score of 0.697 (Fig. 6b). These results support that the TMO-Net model with data pre-training not only demonstrates robustness and generalization across different cancer downstream tasks but also proves effective across diverse forms of cancer multi-omics datasets.

Our study characterized key gene expression features associated with various drug treatments to elucidate the connections between drug responses and cancer molecular profiles. We ranked these gene features based on the importance score and utilized them to identify the Reactome pathway [45] enrichments linked to different drug responses. Notably, most enriched gene pathways, predominantly related to cell cycle pathways, were consistent across all examined drugs (Fig. 6c, Additional file 1: Fig. S3). Subsequently, we explored drug-specific gene pathways. Our findings revealed distinct associations: the response to gemcitabine was linked to the activation of Fc gamma receptor signaling; paclitaxel correlated with tumor metabolism pathways; erlotinib’s effectiveness was connected to cell–cell interaction mechanisms; and cetuximab was associated with lipid metabolism pathways (Fig. 6c). In *EGFR* inhibitor-treated datasets, we revealed the significant correlation between the *EGFR* gene expression and IG values of *EGFR* gene values in the cetuximab but not the erlotinib-treated samples (Additional file 1: Fig. S4). These findings highlight the variability in drug responses across diverse tumor molecular profiles, limited by the size of testing datasets and potential drug resistance; the clinical implications of these results warrant further in-depth discussion.

### TMO-Net enhances prognosis prediction with tumor multi-omics datasets

**Fig. 7:**
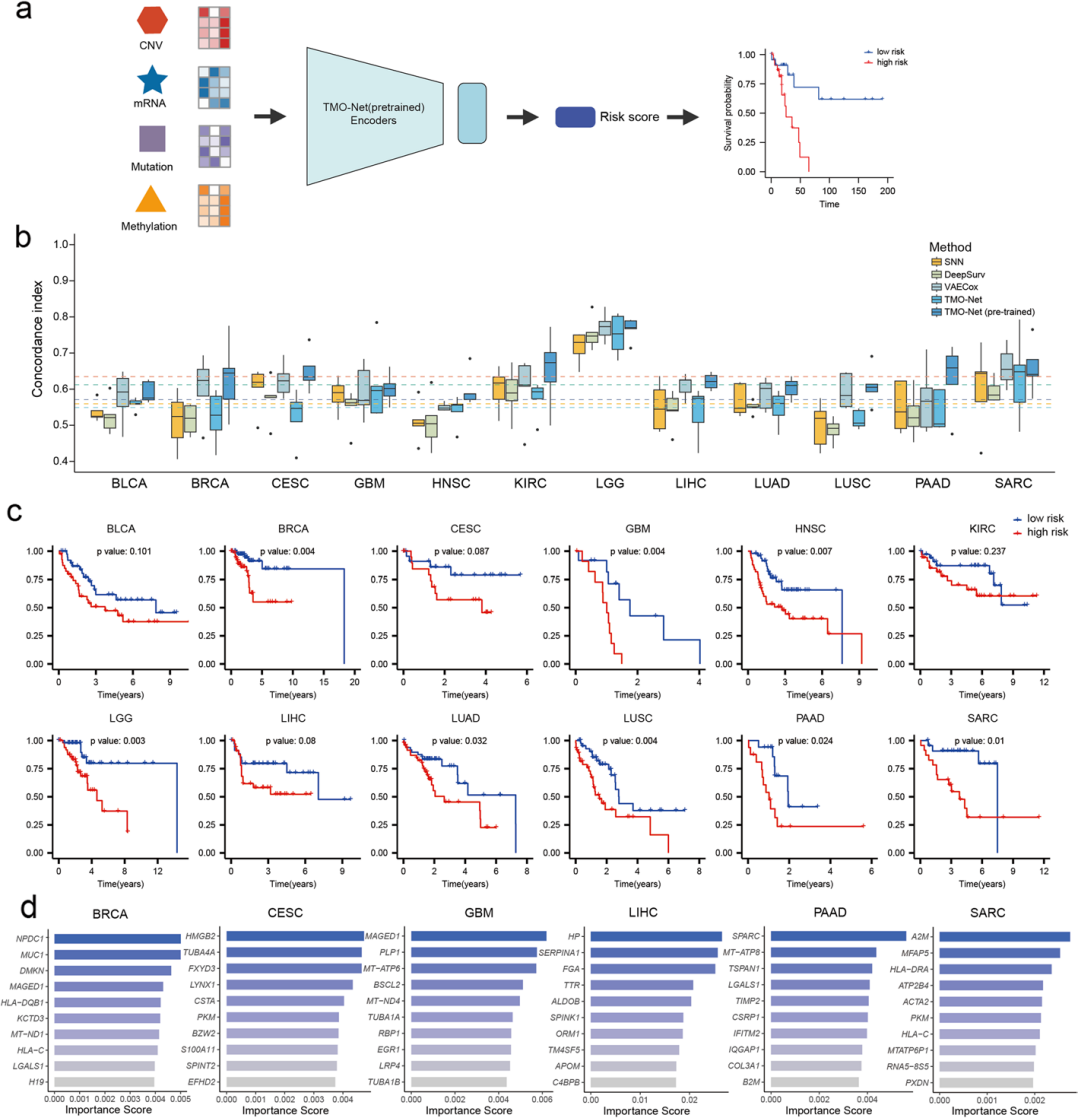
TMO-Net model can predict the prognosis of pan-cancers. **a** Diagram of TMO-Net model adapted for predicting primary or metastatic cancer samples with multi-omics inputs. The predicted risk scores of cancer patients were used to classify high- and low-risk patient groups for downstream survival analysis. **b** Comparison of the performance of different models in predicting patient prognosis of different cancers, the C-index was applied for performance evaluation, and the horizontal line represents the total average C-index across pan-cancers. **c** Kaplan–Meier analysis of the overall survival prediction of low- and high-risk patients stratification by the TMO-Net (pre-trained) model across 12 cancer types. Low- and high-risk patient groups were defined by the predicted median risk scores. The log-rank test was used to test for statistical significance between the predicted prognosis of low- and high-risk cancer patients. **d** Bar plots of important gene expression features most related to the prognosis of identified cancer subtypes

Another major challenge in cancer multi-omics analysis is to accurately characterize the prognosis of cancer patients with distinct molecular features and further benefit the development of personalized treatment design and precision oncology [2]. In this study, we employed the TMO-Net model for predicting the overall survival (OS) status of cancer patients across 12 different subtypes (Fig. 7a). The prognosis prediction model was established with the Cox survival regression network [46] to predict the risk score of individual patients, and the model was trained with weak supervision method. We evaluated the performance of the TMO-Net model using the Concordance index (C-index) score with other baseline models, including SNN, DeepSurv [46], VAECox [47], and the TMO-Net model trained from scratch. The TMO-Net (pre-trained) model achieved the highest average C-index of 0.6344 across all cancers and outperforms all other baseline methods in 11 of 12 cancer subtypes (Fig. 7b). Notably, the TMO-Net (pre-trained) model performs better than TMO-Net model fine-tuned with only single cancer-specific multi-omics dataset, highlighting the advantages of pan-cancer data pre-training on cancer downstream tasks. Furthermore, we conducted an additional experiment aimed at validating the significance of model pre-training in tumor multi-omics datasets. We integrated independent tumor multi-omics datasets from the Clinical Proteomic Tumor Analysis Consortium (CPTAC) project [48], encompassing proteomics, gene expression, gene mutation, and CNV modalities. Compared to the performance of TMO-Net models trained from scratch and pre-trained, we demonstrated that model pre-training substantially improves prognosis prediction in CPTAC multi-omics datasets using the TMO-Net model (Additional file 2: Table S6).

We further evaluated our model’s capabilities to classify cancer patients into high- and low-risk cohorts. Patients were grouped based on the median survival risk scores predicted for individual cancer cohorts. We then visualized patient stratification using Kaplan–Meier survival curves (Fig. 7c) and assessed the differences between high and low-risk groups through Log-rank tests. Our results demonstrate that the TMO-Net (pre-trained) model effectively distinguishes between high- and low-risk cancer patients in 8 out of 12 cancer cohorts (*p* < 0.05). In addition, we employed data interpretation algorithms to validate TMO-Net’s proficiency in identifying crucial genomic features for patient survival prediction. We calculated gene expression importance scores for various cancer subtypes (Fig. 7d, Additional file 1: Fig. S5). Commonly shared features across most cancer subtypes included mitochondrial genes, HLA-related genes, and keratin family genes, known for their roles in tumor metabolism, progression, and immune responses [49–53]. Furthermore, we revealed other cancer subtype-specific prognostic gene expression features, including *MFAP5* [54], *SPARC* [55], *BSCL2* [56], *SERPINA1* [57], *S100A11* [57], and *MUC1* [58], among others (Fig. 7d). These results affirm TMO-Net’s efficacy in extracting biologically informed genomic features from multi-omics tumor datasets for various applications, thereby enhancing our understanding of cancer pathogenesis and potentially expanding its use in clinical settings.

## Discussion

The complex and heterogeneous nature of tumor pathogenesis necessitates a deeper understanding of its molecular mechanisms and the resulting clinical phenotype changes [59]. Although multi-omics profiling of tumor samples offers a multifaceted view of cancer development, challenges in modality fusion and model interpretation often limit our complete understanding of these datasets [2]. The emergence of foundation models in the biomedical field [18] opens new opportunities for creating algorithms that address various clinical challenges in oncology. In this study, we introduced a multi-omics tumor data fusion framework named TMO-Net, designed for pre-training on extensive tumor multi-omics datasets and enabled for different downstream tasks with incomplete data modalities. Using self-supervised learning, the TMO-Net model effectively generates biologically significant joint embeddings from multi-omics datasets, providing enhanced representation of diverse tumor samples. Furthermore, the pre-trained TMO-Net model can be adapted to different downstream tasks, such as drug response and patient prognosis prediction, especially in scenarios with limited data. Our results demonstrated that the pre-trained TMO-Net model surpasses its counterparts trained from scratch, proposing the potential of large-scale data pre-training and foundation model approach in future tumor multi-omics analyses.

Compared with other tumor multi-omics data fusion algorithms, the TMO-Net model integrates a “Cross Fusion Module” network. This network leverages the Product-of-Expert algorithm to efficiently learn joint latent embeddings from multi-omics inputs, enabling the processing of incomplete data modalities. The model’s self-supervised training approach facilitates learning of feature interactions across various modalities, thereby enhancing its ability to generalize and adapt to new datasets and tasks. Additionally, TMO-Net’s proficiency in identifying important omics features across different clinical outcomes aids in understanding key genomic regulations during various tumor development stages and their clinical relevance. These capabilities position TMO-Net as a versatile and adaptable framework, well-suited for exploring tumor multi-omics datasets and addressing a broad range of downstream tasks in tumor research.

Although the TMO-Net model performed well in various tumor downstream applications, our study has various limitations. The primary challenge is the scarcity of large-scale paired multi-omics datasets in oncology, which are crucial for effective model pre-training. Additionally, the diverse data preprocessing methods used in biomedical datasets complicate comprehensive learning across individual modalities. The restriction of paired multi-omics datasets also complicates the usage of mass-published biomedical datasets and further applications of our models. Furthermore, the different modalities of tumor research follow its underlying biological roles as the “Central Dogma,” future fusion frameworks should incorporate the causality and connections between different omics features or integrate the advanced biological regulation databases to better characterize the development and progression of tumors. Furthermore, in addition to understanding the significance of distinct molecular features for model outcomes, recognizing regulatory roles across diverse omics features through self-supervised learning is equally crucial [29, 60, 61]. By discerning the regulatory associations within molecular features across varying scales, we can uncover more intricate mechanisms of tumorigenesis and development. The fusion of biological insights with interpretable model frameworks presents a promising pathway for enhancing the construction of multi-omics cancer deep models in further research.

## Conclusions

The TMO-Net model paves the way for harnessing the power of established multi-omics data in cancer research. By demonstrating its effectiveness on diverse tumor datasets and downstream tasks, TMO-Net proves that pre-training on multi-omics datasets can significantly enhance representation learning, both for multi-omics and incomplete omics data. Furthermore, its ease of transferability to other omics-based tumor research applications adds its value in oncology. This study lays the groundwork for developing future multi-omics foundation models that will accelerate cancer research.

## Methods

### Data preprocessing

We sourced multi-omics data for 32 pan-cancers from the TCGA database, which consisted of 8174 samples, including normal tissue samples. Each cancer subtype dataset incorporated four types of omics data: RNA-Seq gene expression profiles, DNA methylation profiles, gene mutation data, and copy number variation (CNV) data. Additionally, corresponding clinical information was included. The multi-omics data preprocessing procedures and criteria for feature selection were followed as in Chiu et al. [19]. In brief, the gene expression data, represented as log2(TPM + 1), underwent filtering to exclude genes with standard deviation (SD) greater than 1, resulting in a final set of 6016 genes. In gene mutation profiles, genes were filtered to include only those mutated in at least 1% of TCGA samples, resulting in 4539 genes for further analysis. In DNA methylation analysis, probes that exhibited low methylation (beta-value < 0.3) in over 90% of TCGA samples were removed, leaving 6617 features for subsequent analysis. Copy number variation (CNV) analysis was conducted by filtering 7460 informative segments based on criteria including fewer than 5% of samples with zeros, a mean of absolute values exceeding 0.20, and a coefficient of variation greater than 0.20 across all samples. The multi-omics profiling and clinical information in other downstream fine-tuning tasks are also available from public sources, and the pre-processed multi-omics datasets used in other studies were obtained. In model fine-tuning, intersected omics features with the pre-training multi-omics TCGA datasets were used. The independent CPTAC multi-omics datasets were obtained, including proteomics data in 7970 features, gene expression data in 39,451 dimensions, gene mutation data in 1553 genes, and copy number variation (CNV) data in 60,604 segments from four tumor subtypes (COAD, GBM, LUAD, and GBM).

### TMO-Net framework

The TMO-Net model contains a hybrid architecture of self- and cross-modal variational autoencoders (Fig. 1b, c). The self-modal variational autoencoders are designed to capture omics-specific representations and reconstruct themselves, while the cross-modal variational autoencoders focus on learning the cross-omics associations and predicting the target omics from others. Notably, a “Cross Fusion Module” was designed to ensure that with the absence of certain omics data, it can generate approximate patient-level latent embeddings from other available omics and can be further utilized in different downstream tasks. The discriminator and contrastive losses were designed to enhance the performance of cross-modal imputation and representation learning of pan-cancer multi-omics datasets. In the model pre-training phase, paired multi-omics datasets were required to learn all the self- and cross-modal associations. In the model fine-tuning phase, the self-autoencoders of available data modalities were updated and the cross-autoencoders of missing modalities were locked for cross-modal inference; the generated joint embeddings were used for training task-specific neural networks.

### Self-modal variational autoencoder

Following the basic framework of the variational autoencoder, the self-modal variational autoencoder (self-VAE) was designed to capture omics-specific representations and reconstruct the target omics. For multi-omics data, each omics data is first passed into a self-VAE encoder to reduce the dimensionality from high dimensionality to 64 in the embedding space. For the input **x** of omics *k*, the self-VAE encoder network outputs two vectors, the mean vector $\mu$ and the standard deviation vector $\delta$, which determined the Gaussian distribution $N(\mu ,\delta )$ of the latent variable **z** in the embedding space. The latent variable **z** is randomly sampled from the distribution. To achieve backpropagation in the deep neural network, the reparameterization trick was applied using Eq. (1) to approximate the latent variable **z**:

1 $$z=\mu +\delta \varepsilon$$

Then, the latent variable **z** was passed to reconstruct the omics data $\overline{x}$ of the input $x$ through the VAE decoder network. The self-VAE network was optimized by maximizing the variational evidence lower bound (ELBO) defined in Eq. (2):

2 $${\text{ELBO}}_{\text{self}}={E}_{({q}_{\phi }(z|x))}[-log{q}_{\phi }(z|x)+log{p}_{\theta }(x,z)]$$

Equation (2) can further transform into Eq. (3):

3 $${\text{ELBO}}_{\text{self}}={E}_{(z\sim {q}_{\phi }(z|x))}log{p}_{\theta }(x\mid z)-{D}_{KL}({q}_{\phi }(z|x)\parallel {p}_{\theta }(z))$$

The network was optimized to maximize Eq. (3), and for the deep learning model, the generative distributions can be implemented by the deep decoder neural network, and the variational posteriors by deep encoder neural networks. The self-VAE loss can be defined as follows in Eq. (4):

4 $${\mathcal{L}}_{\text{self}}=MSE({x}_{j},\overline{{x}_{j}})+{D}_{KL}({q}_{\phi }(z\mid x)\parallel {p}_{\theta }(z))$$

### Cross-modal variational autoencoder

The cross-modal variational autoencoder (cross-VAE) is devised to learn the cross-omics associations and predict target omics from others. For the target omics *k,* the input **x** of other omics was passed into encoders of cross-VAE to reduce the high dimensionality to 64. The encoder of cross-VAE generates two vectors, the mean vector $\mu$ and the standard deviation vector $\delta$, which determined the variational posteriors Gaussian distribution $N(\mu ,\delta )$ of the latent variable **z** in the embedding space, and represented as the inferred latent embedding of omics *k* from others, which was defined in Eq. (5):

5 $$q({z}_{k}\mid {x}_{{k}{\prime}})({k}{\prime}\ne k)$$

### Cross Fusion Module

To get a better and full multi-omics representation with missing modalities data, we designed a “Cross Fusion Module” to fuse embeddings of all other encoders of cross-VAE into a common space. We employed a product-of-experts (PoE) module to obtain the product of the marginal variational posteriors from all existing modalities into a joint variational posterior as follows:

6 $${q}_{\phi }({z}_{k}\mid {x}_{{\nu }^{k}})=\prod {q}_{\phi }({z}_{k}\mid {x}_{{k}{\prime}})({k}{\prime}\in {\nu }^{k},k\notin {\nu }^{k})$$

The joint cross-modal ELBO can then be defined as:

7 $${\mathcal{L}}_{\text{cross}-\text{elbo}}={E}_{{q}_{\phi }({z}_{k}\mid {x}_{{\nu }^{k}})}log{p}_{\theta }({x}_{k}|{z}_{k})-{D}_{KL}({q}_{\phi }({z}_{k}|{x}_{{\nu }^{k}})\parallel p({z}_{k}))({k}{\prime}\in {\nu }^{k},k\notin {\nu }^{k})$$

In this way, we can employ the observed modalities ${x}_{{\nu }^{k}}$ to infer the latent embedding of ${x}_{k}$, further reconstructing missing modal ${x}_{k}$. We also utilized the KL-Divergence to align distributions between self-variational posteriors and cross joint-variational posteriors as follows:

8 $${{\mathcal{L}}_{\text{cross}-\text{kl}}=D}_{KL}({q}_{\phi }({z}_{k}|{x}_{{\nu }^{k}})\parallel {q}_{\phi }(z\mid x))$$

The parameter $\theta$ of the omics-specific decoder in cross-VAE was consistent with the decoders of the self-VAE, and the cross-modal loss can be computed as:

9 $${\mathcal{L}}_{\text{cross}}={\lambda }_{\text{cross}-\text{elbo}}{\mathcal{L}}_{\text{cross}-\text{elbo}}+{\lambda }_{\text{cross}-kl}{\mathcal{L}}_{\text{cross}-kl}$$

The ${\lambda }_{\text{cross}-\text{elbo}}$ and ${\lambda }_{\text{cross}-\text{kl}}$ are the hyperparameters to balance the weight of different loss functions.

### Latent embedding fusion

For omics *k*, we get two latent representations, the ${z}_{{k}_{self}}$ generated by self-VAE and ${z}_{{k}_{cross}}$ generated by joint cross-VAEs from other modalities. To get embedding from full modalities, we fuse these two embeddings as the joint latent representation for the omics *k*, as follows:

10 $${z}_{{k}_{\text{fusion}}}={z}_{{k}_{\text{self}}}\oplus {z}_{{k}_{\text{cross}}}$$

While omics *k* is missing, we can also get the representation from the observed modalities as follows:

11 $${z}_{{k}_{fusion}}={z}_{{k}_{cross}}$$

Finally, embeddings of all modalities were concatenated to represent the sample-level multi-modal representations for downstream model prediction and fine-tuning.

### Discriminator

Discriminators assist in improving the generative capacity of TMO-Net during the pre-training stage [62]. The discriminator of modality *k,*$Di{s}_{k-s}$ takes either original data ${x}_{k}$ or reconstructed data ${\widetilde{x}}_{k-s}$ as input and outputs a label represents data sources. The discriminator $Di{s}_{k-c}$ takes ${\widetilde{x}}_{k-c}$ to predict the source modal used in cross-encoders. Consequently, the self- and cross-encoders of TMO-Net were trained adversarial to deceive the modality discriminators. The discriminator loss was defined as:

12 $${\mathcal{L}}_{dis}=CE(Di{s}_{k-s}\left({\widetilde{x}}_{k-s}\right),0)+CE(Di{s}_{k-s}\left({x}_{k}\right),1)+CE(Di{s}_{k-c}\left({\widetilde{x}}_{k-c}\right),k)$$

$CE\left(\cdot ,\cdot \right)$ represents the cross-entropy loss.

### Contrastive learning

To further enhance representation learning of pan-cancer multi-omics datasets, we applied the contrastive learning [63] strategy to obtain a latent feature space with enhanced pan-cancer supervision. In the training batch, for target sample ${x}_{t}\in {X}_{t}$ in a specific cancer subtype *t*, the positive samples set is ${X}_{t}$, as well as their latent embeddings ${Z}_{t}$. In contrast, the latent embeddings ${Z}_{n}$ from other cancer subtypes were defined as negative samples [64]. The contrastive loss was then designed to increase the latent distance similarity across positive samples ${\mathcal{L}}_{pos}$ and reduce that in negative samples ${\mathcal{L}}_{neg}$, which were defined as:

13 $${\mathcal{L}}_{pos}=\frac{\sum_{z_{t},z^{\prime}_{t}\epsilon Z_{t}}\text{sim}(z_t,z_t^\prime)/\tau}{\sum_{z_{t},z^{\prime}_{t}\epsilon Z_t}\mathbb{1}(z_t,z^\prime_t)/\tau},\quad{\mathcal{L}}_{neg}=\frac{\sum_{z_{t}\epsilon Z_{t},z_{n}\epsilon z_{n}}[1-\text{sim}(z_t,z_n)]/\tau}{\sum_{z_{t}\epsilon z_{t},z_{n}\epsilon z_{n}}\mathbb{1}(z_t,z_n)/\tau}$$

$\text{sim}\left(\cdot ,\cdot \right)$ represents the similarity calculation function, $1\left(\cdot ,\cdot \right)$ represents a counting function that returns 1 if both inputs are not empty. $\tau$ is the temperature parameter used to control the strength of the distance constraints. Finally, the contrastive learning loss is obtained by the linear combination of the positive sample pair loss and the negative sample pair loss as defined in Eq. (14).

14 $${\mathcal{L}}_{con}= {\lambda }_{pos}{\mathcal{L}}_{pos}+ {{\lambda }_{neg}\mathcal{L}}_{neg}$$

### Model pre-training loss

The TMO-Net model is jointly optimized by four losses during the pre-training stage: (1) self-modal ELBO ${\mathcal{L}}_{self}$ of self-VAE; (2) cross-modal ELBO ${\mathcal{L}}_{\text{cross}}$ of cross-VAE and KL divergence of original and inferred variational posteriors; (3) adversarial loss of discriminators ${\mathcal{L}}_{dis}$; (4) contrastive loss ${\mathcal{L}}_{con}$. The overall pre-training loss optimization was defined as follows:

15 $$\text{Min }{(\lambda }_{self}{\mathcal{L}}_{self}+{\lambda }_{cross}{\mathcal{L}}_{cross}+{\lambda }_{con}{\mathcal{L}}_{con})$$

16 $$\text{Max }( {\lambda }_{dis}{\mathcal{L}}_{dis})$$

${\lambda }_{*}$ are the weight coefficients for corresponding losses. We utilized various hyperparameter configurations to achieve a balance in weighting different loss functions during the optimization in model pre-training. The specific hyperparameters employed in the pre-training phase are detailed in Table S7, and we conducted loss ablation experiments to assess the significance of individual losses, as summarized in Table S8.

### TMO-Net pre-training process

In the model pre-training process, the TCGA multi-omics data were split into fivefold by cancer types for cross-validation. We applied the Adam optimizer with learning rate 1e − 4 and weight decay 5e − 4 for the discriminator part and used the Adam optimizer with learning rate 1e − 5 and weight decay 1e − 4 descent by epoch. TMO-Net was pre-trained for a maximum of 100 epochs with early stopping to avoid overfitting. We fixed all random seeds during model initialization and training to ensure the reproducibility of the training process. To improve the ability of cross-modal reconstruction, the strategy for modality masking is randomly generating the integer number from 0 to 3, corresponding to the mask or non-mask status of the four modalities of TCGA data. During the pre-training state, the LogME [65] method is used to estimate the cancer-type clustering score for the best model selection.

### Downstream task model fine-tuning

During the fine-tuning phase for downstream tasks, we formulated oncological and clinical objectives, which encompassed pan-cancer subtype classification, breast cancer subtype classification, cancer metastasis prediction, drug response prediction, and pan-cancer survival prediction. Leveraging the multi-omics cancer representations generated by TMO-Net, a feedforward neural network served as the classifier to produce outcome predictions. For pan-cancer subtype classification, we kept the parameters of TMO-Net frozen, updating only the downstream classifier during the fine-tuning process to assess the sample representation performance of pre-trained TMO-Net. In contrast, for other downstream tasks, both the parameters of TMO-Net and the downstream classifier were updated to acquire informative representations supervised by the task loss function. For instance, for missing modalities in the fine-tuning datasets, the cross-inference variational encoders of missing modalities in the TMO-Net model remained frozen to maintain the quality of cross-modal reconstruction. In model fine-tuning, we partitioned the training multi-omics dataset into fivefolds for cross-validation and utilized an external validation dataset for performance evaluation. We utilized the Adam optimizer with a learning rate of 1e − 5 and weight decay of 1e − 4, descending by epoch with early stopping to prevent overfitting. Additionally, apart from the loss associated with the classification task, other losses from the pre-training stage were disregarded.

For the multi-label classification task, we applied a C-dimensional one-hot vector to represent the probability of the sample in the $\text{C}$ class and proposed the output prediction result is $\widehat{\text{y}}=\left[\widehat{{\text{y}}_{1}},\cdots ,\widehat{{\text{y}}_{\text{C}}}\right]$, the cross-entropy loss was defined as follows:

17 $${\mathcal{L}}_{\mathcal{C}\mathcal{E}}=-{\sum }_{i=1}^{c}{y}_{i}log \left(Softmax\left({\widehat{y}}_{i}\right)\right)$$

For the survival prediction task, the clinical information of TCGA datasets was utilized and the model was fine-tuned with full or incomplete omics. Additionally, we filtered pan-cancer subtypes with more than 50 uncensored samples and finally included 12 cancer subtypes for performance evaluation. The Adam optimizer is used to optimize the loss function Cox loss and reconstruction loss. The learning rate is set to 1e − 5, and the model freezing layer setting is consistent with the classification task. For model validation in the survival prediction task, we involved an independent tumor multi-omics dataset from the CPTAC project and included 4 tumor subtypes for performance evaluation. The Cox proportional hazards [66] and maximum likelihood (ML) model were used for evaluating the hazard ratio of the patient’s prognosis, assuming the output of the cross-modal fusion as:

18 $${h}_{\uptheta }=\text{l}\left(\text{concatenate}\left({{z}_{m}}_{m=1}^{M}\right)\right)$$

where $\text{l}$ was the MLP for the low-rank multimodal fusion output, ${h}_{\uptheta }$ was hazards of multi-omics samples. The Cox likelihood was constructed by the observed order of events named “partial” likelihood as described in [67–69]:

19 $${\mathcal{L}}_{part}=\prod_{i:{E}_{i}=1}\frac{{e}^{\widehat{{h}_{\theta }}({x}_{i})}}{{\sum }_{j\in R({T}_{i})}{e}^{\widehat{{h}_{\theta }}({x}_{j})}}$$

where $\text{i},\text{j}$ are the sample numbers, and $\text{E}=1$ represents the termination event (right censored) that occurs. $\text{R}\left({T}_{i}\right)$ means that ${T}_{j}>{T}_{i}$ is satisfied, and the corresponding Cox loss (defined as neg log partial likelihood loss) is calculated by the maximum likelihood of ${\mathcal{L}}_{part}$ [70]:

20 $${\mathcal{L}}_{cox}=-\text{log}{\mathcal{L}}_{part}=-\sum_{i:{E}_{i}=1}\left(\widehat{{h}_{\theta }}\left({x}_{i}\right)-\text{log}{\sum }_{j\in R\left({T}_{i}\right)}{e}^{\widehat{{h}_{\theta }}\left({x}_{j}\right)}\right)$$

### Implementation of baseline methods

We introduced a set of baseline methods for evaluating the performance of our TMO-Net model, encompassing OmiVAE [11] for pan-cancer multi-omics learning, Moanna for breast cancer subtype classification [31], CVAE for cancer metastasis prediction [39], MOLI for drug response prediction [44], and VAECox [47], SNN, and DeepSurv [46] for survival prediction. All baseline models were implemented following methodologies outlined in published literature and utilizing corresponding code repositories available in public domains. All training procedures maintained consistent hyperparameter settings and employed consistent dataset separation of multi-omics datasets to ensure fair comparisons across different methods and tasks.

### Evaluation metric

In the model pre-training phase, the LogME method was used for checkpoint selection. In the cross-modal inference task, Pearson’s correlation coefficients and coefficient of determination (*R*^*2*^) were applied to quantify the performance in gene expression imputation from other modalities. The area under the curve (AUC) method was used to compare the performance of gene mutation prediction from other modalities. In classification tasks, we utilized accuracy, precision, recall, and F1-score for performance evaluation. The C-index, Kaplan–Meier survival curves, and Log-rank tests were utilized to characterize the performance of survival analysis tasks.

### Model interpretation analysis

The integrated gradients (IG) method was utilized for model interpretation analysis, which assesses feature importance by integrating gradients along a path from actual input to the baseline value across individual features. Let ${f}_{\widehat{\theta }}\left(x\right)$ represents the output of the pre-trained TMO-Net model for the omics input $x$, and $\widehat{\theta }$ be model parameters, and $\nabla {f}_{\widehat{\theta }}\left(x\right)$ represents the gradient of the model’s output to the input . The integrated gradient value ${IG}_{i}\left(x\right)$ for the ${i}^{\text{th}}$ feature was computed as follows:

21 $${IG}_{i}\left(x\right)=\left({x}_{i}-{x}_{i}{\prime}\right)\times {\int }_{\alpha =0}^{1}\nabla {f}_{\widehat{\theta }}({x}{\prime}+\alpha \times \left(x-{x}{\prime}\right))d\alpha$$

where and ${x}_{i}{\prime}$ is the ${i}^{\text{th}}$ actual input and the baseline input. $\alpha$ is the parameter varying from 0 to 1. For each molecular feature $g$, we computed its averaged absolute IG value within a specific sample group $S$, as the contribution value of $g$ in specific downstream tasks, which was defined as follows:

22 $$\text{I}\left(g\right)=\frac{\sum_{x \in S}\left|{IG}_{g}\left(x\right)\right|}{|S|}$$

For the classification task, the class-specific neurons in the SoftMax layer were selected as model output, as well as the hazard ratio of patient samples in the survival prediction task. To evaluate the contributions of individual modalities in model outcomes, the sum of the IG values for all molecular features within individual modalities was used. We iterated this process within different downstream tasks and ranked molecular features related to different model outcomes for subsequent biology interpretation and pathway analyses.

### Implementation details

All experimental procedures were conducted on a workstation equipped with two Intel Xeon Silver 4210R CPUs and four NVIDIA GeForce RTX 3090 GPUs. The TMO-Net model was constructed based on the Python platform and Pytorch library. The model interpretation analysis was implemented using the Captum package. Throughout all benchmarks and experimental scenarios, we adhered to the utilization of default hyperparameters, unless otherwise specified explicitly.

### Supplementary Information

Additional file 1: Supplementary figures in TMO-Net research.

Additional file 2: Details of pre-training and task performance of TMO-Net.

Additional file 3: Review history.

## Data Availability

The pre-training and fine-tuning datasets of this study are all available online from public studies. The pre-processed TCGA pan-cancer datasets for pre-training were obtained from: https://codeocean.com/capsule/7914207/tree/v1 [71]. The multi-omics dataset of breast cancers is available at 10.5281/zenodo.4326602 [72]. The pre-processed data used for metastasis prediction is available at https://github.com/SomayahAlbaradei/MetaCancer [73]. The multi-omics profiles and drug response datasets from GDSC for cell line drug response prediction are retrieved from https://www.cancerrxgene.org/downloads/anova [74]. The publicly available multi-omics dataset for PDX Encyclopedia dataset for external validation is available in the Supplementary Table 1 of Gao et al. [75]. The processed multi-omics profiles and clinical information of the CPTAC cancer datasets can be downloaded at the “Data Source” section of https://www.linkedomics.org/ [76], including the data of RNAseq (Gene level, Tumor), CNV (log2 ratio), Mutation (Gene level), Proteomics (Tumor), and Survival from individual cancers. The source code of the TMO-Net model is available online: https://github.com/FengAoWang/TMO-Net [77] and is published under the MIT license. All processed datasets used for TMO-Net model pre-training and fine-tuning are deposited in Zenodo with the accession code of https://zenodo.org/records/11258239 [78].
