# Supplementary material for: TMO-Net: an explainable pretrained multi-omics model for multi-task learning in oncology
Source: Genome Biol. 2024 Jun 6;25:149. doi: 10.1186/s13059-024-03293-9 (PMC11157742; doi:10.1186/s13059-024-03293-9)
Supplement: Supplementary file 1 — Additional file 1: Supplementary figures in TMO-Net research. [file 13059_2024_3293_MOESM1_ESM.docx]

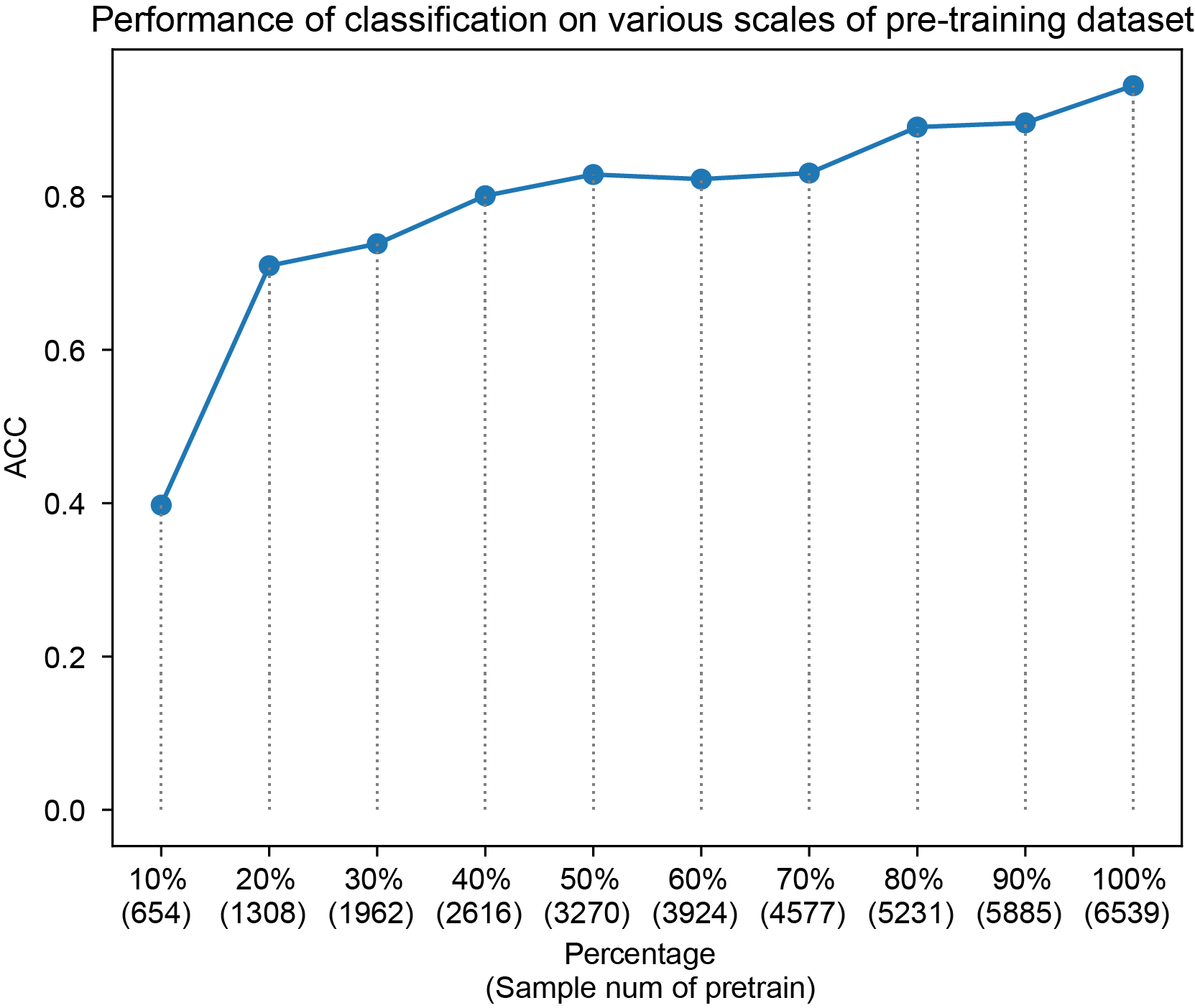


Fig S1. Comparison of the performance of cancer subtype classification task with different scales of pre-training multi-omics dataset.


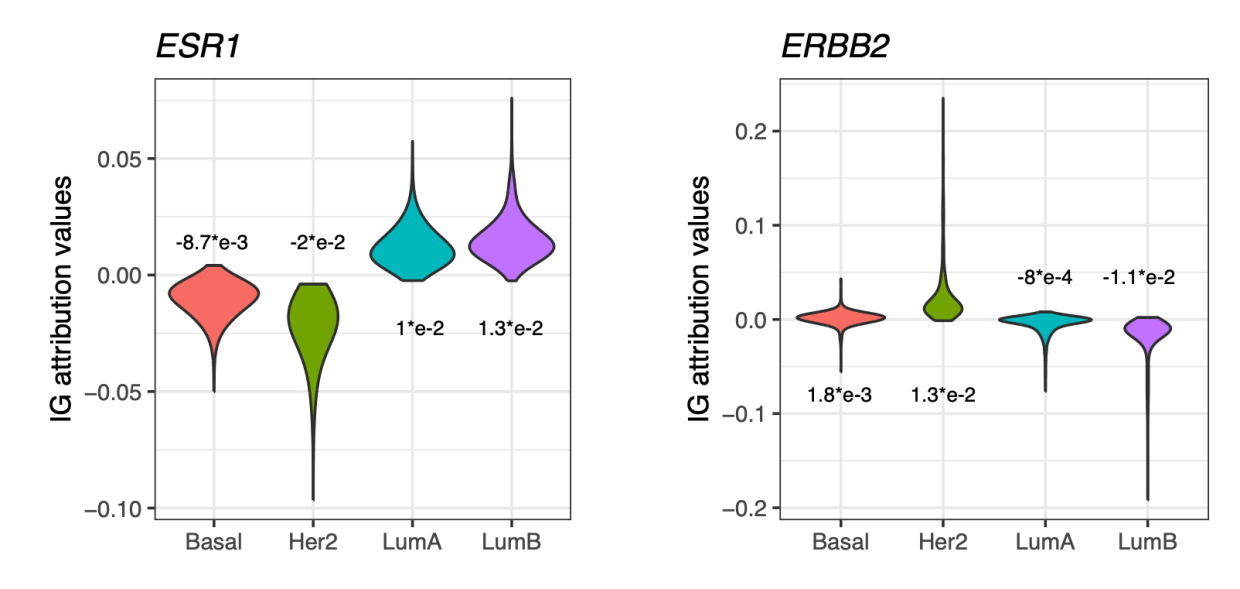
Fig S2. Comparison of the performance of cancer subtype classification task with different scales of pre-training multi-omics dataset.


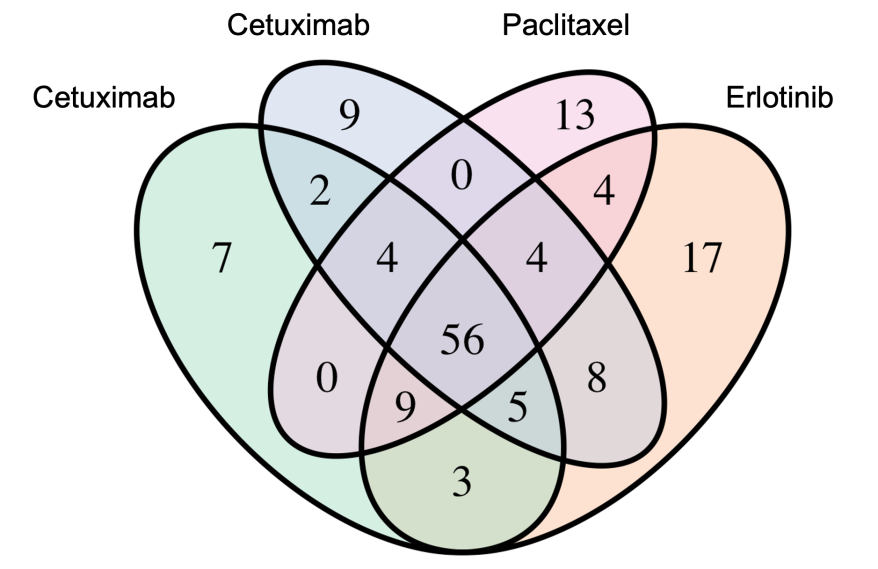


Fig S3. The overlap of pathway enrichments across different drug treatments.


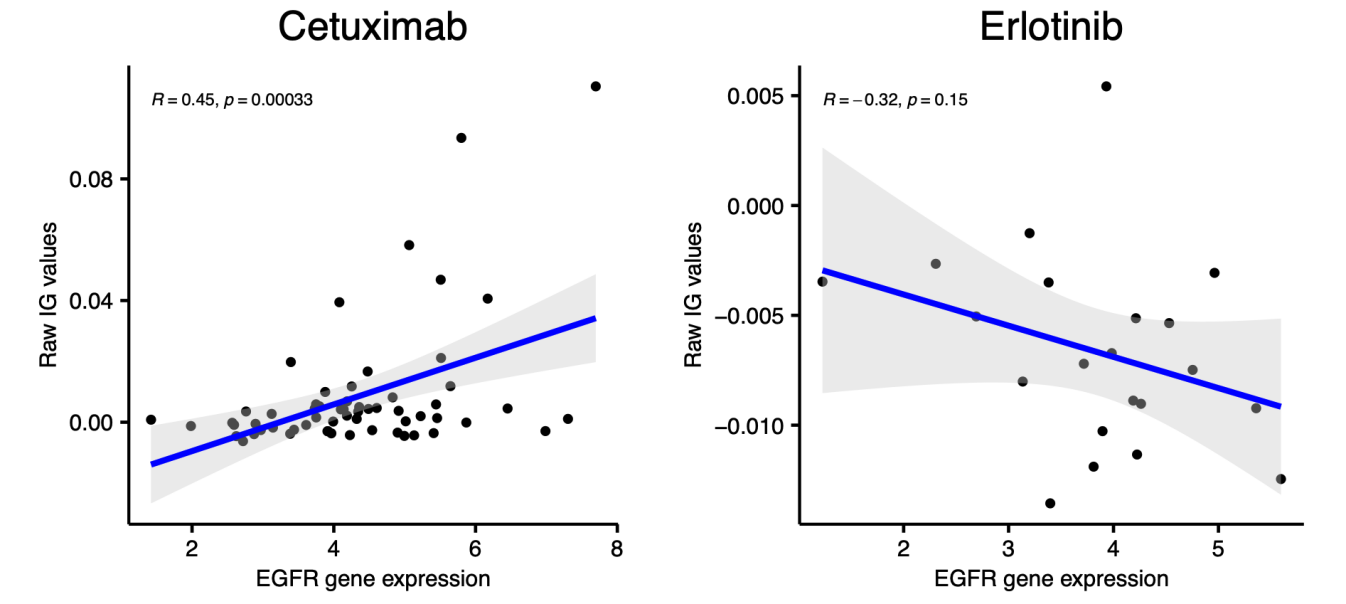


Fig S4. Correlation analyses of the *EGFR* expression values and their IG values in different *EGFR* inhibitors.


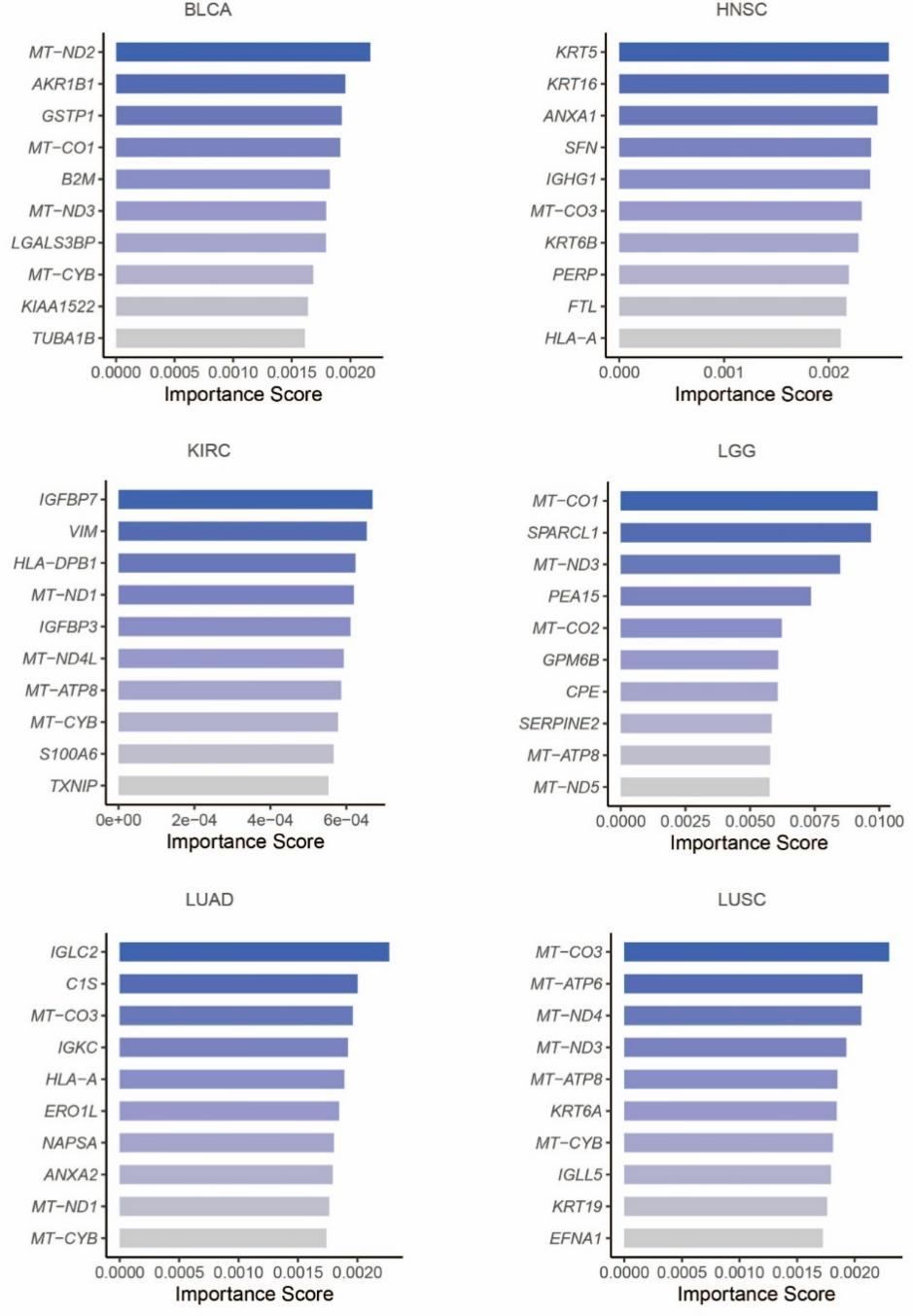


Fig S5. The important gene expression features related to prognosis for various cancer types.
