# Supplementary material for: TMO-Net: an explainable pretrained multi-omics model for multi-task learning in oncology
Source: Genome Biol. 2024 Jun 6;25:149. doi: 10.1186/s13059-024-03293-9 (PMC11157742; doi:10.1186/s13059-024-03293-9)
Supplement: Supplementary file 2 — Additional file 2: Details of pre-training and task performance of TMO-Net. [file 13059_2024_3293_MOESM2_ESM.docx]

Table S1. Performance comparison of pan-cancer subtype classification, classified by XGBoost model.

| Multi-omics input | | | | |
| --- | --- | --- | --- | --- |
| Method | ACC | Precision | recall | F1 score |
| TMO-Net | **0.792** | **0.783** | **0.802** | **0.751** |
| OmiVAE | 0.719 | 0.750 | 0.644 | 0.636 |

| Only mRNA expression input | | | | |
| --- | --- | --- | --- | --- |
| Method | ACC | Precision | recall | F1 score |
| TMO-Net | **0.806** | **0.758** | **0.736** | **0.733** |
| OmiVAE | 0.720 | 0.748 | 0.623 | 0.617 |

Table S2. Contributions of pre-training data scales in the pan-cancer classification task, classified by classification network.

| Data size | ACC | Precision | Recall | F1 score |
| --- | --- | --- | --- | --- |
| 654 (10%) | 0.3975 | 0.2239 | 0.2353 | 0.1989 |
| 1308 (20%) | 0.7097 | 0.5582 | 0.5446 | 0.5242 |
| 1962 (30%) | 0.7383 | 0.6776 | 0.5997 | 0.5942 |
| 2616 (40%) | 0.8009 | 0.7219 | 0.6842 | 0.6786 |
| 3270 (50%) | 0.8286 | 0.7550 | 0.7097 | 0.7005 |
| 3924 (60%) | 0.8226 | 0.7643 | 0.7627 | 0.7567 |
| 4577 (70%) | 0.8305 | 0.7683 | 0.7581 | 0.7546 |
| 5231 (80%) | 0.8905 | 0.8487 | 0.8264 | 0.8310 |
| 5885 (90%) | 0.8960 | 0.8316 | 0.8214 | 0.8138 |
| 6539 (100%) | **0.9445** | **0.8927** | **0.8817** | **0.8819** |

Table S3. Contributions of different modalities in the pan-cancer classification task

| Cancer Type | gene expression | DNA methylation | gene mutation | copy number variation (CNV) |
| --- | --- | --- | --- | --- |
| ACC | 4.184484703 | 5.517831275 | 0.066589384 | 0.210567966 |
| BLCA | 4.437674079 | 5.232496711 | 0.334054839 | 0.135867182 |
| CESC | 4.35800042 | 5.216562296 | 0.058221816 | 0.16528564 |
| CHOL | 4.427356239 | 5.13624715 | 0.067397213 | 0.078667939 |
| COAD | 4.401682124 | 5.131295069 | 0.052219773 | 0.124388125 |
| DLBC | 4.239868785 | 5.470517208 | 0.037125865 | 0.088048783 |
| ESCA | 4.609513871 | 5.081509663 | 0.01394069 | 0.158046025 |
| GBM | 4.096903024 | 4.589240368 | 0.033438703 | 0.19819148 |
| HNSC | 4.315672131 | 5.10903255 | 0.031113458 | 0.214965488 |
| KICH | 4.214348759 | 5.143230883 | 0.028312496 | 0.126080668 |
| KIRC | 4.531865731 | 5.116248582 | 0.021413437 | 0.213448106 |
| KIRP | 4.237678491 | 4.872835933 | 0.044965904 | 0.153029751 |
| LGG | 4.389836642 | 4.982489999 | 0.060528383 | 0.185041886 |
| LIHC | 4.30289782 | 4.970509655 | 0.041678019 | 0.169126942 |
| LUAD | 4.304432749 | 5.224168475 | 0.02495416 | 0.106891649 |
| LUSC | 3.939880564 | 5.439625999 | 0.018476779 | 0.172905915 |
| MESO | 4.291522628 | 5.272573557 | 0.017802219 | 0.058097735 |
| OV | 4.532025793 | 4.784307117 | 0.030644862 | 0.083449735 |
| PAAD | 4.089159245 | 4.871647006 | 0.064007792 | 0.146897458 |
| PCPG | 4.129293588 | 4.65820761 | 0.047535296 | 0.170220906 |
| PRAD | 4.594854049 | 5.228774306 | 0.084154532 | 0.116222997 |
| READ | 4.074443706 | 5.240295703 | 0.093657133 | 0.06777761 |
| SARC | 4.158341648 | 4.922763834 | 0.051513842 | 0.168863694 |
| SKCM | 4.369942092 | 4.863415831 | 0.168784381 | 0.185336623 |
| STAD | 4.319955737 | 5.257354216 | 0.022483888 | 0.089889297 |
| TGCT | 4.112766208 | 4.666435804 | 0.063157297 | 0.075808525 |
| THCA | 3.93166193 | 4.772822791 | 0.050800902 | 0.195134507 |
| THYM | 4.183466653 | 4.680707197 | 0.048143491 | 0.226514558 |
| UCEC | 3.734388844 | 4.64334665 | 0.340907368 | 0.096524855 |
| UCS | 4.115386318 | 4.588737961 | 0.051243415 | 0.13184302 |
| UVM | 4.192120043 | 5.068121137 | 0.173226445 | 0.11884854 |
| BRCA | 4.130648826 | 5.205548652 | 0.062417908 | 0.144165628 |

Table S4. Comparison of different pre-training datasets in the PAM50 subtype classification task

| Models | ACC | Precision | Recall | F1-Score |
| --- | --- | --- | --- | --- |
| TMO-Net  (without TCGA-BRCA) | 0.8481 | 0.8526 | 0.8481 | 0.8590 |
| TMO-Net  (with TCGA-BRCA) | **0.8860** | **0.8901** | **0.8860** | **0.8901** |

Table S5. Performance comparison of metastasis prediction across different methods

| Methods | ACC | Precision | Recall | F1 score |
| --- | --- | --- | --- | --- |
| SVM | 0.7444 | 0.8346 | 0.8000 | 0.8148 |
| CVAE | 0.8350 | 0.9040 | 0.8464 | 0.8788 |
| TMO-Net  (from scratch) | 0.8397 | **0.9193** | 0.8677 | 0.8800 |
| TMO-Net  (pre-trained) | **0.8598** | 0.9164 | **0.8821** | **0.8980** |

Table S6. Validation of the TMO-Net model using independent CPTAC datasets

| Cancer subtype | TMO-Net  (pre-trained) | TMO-Net  (trained from scratch) |
| --- | --- | --- |
| COAD | **0.7973** | 0.6060 |
| GBM | **0.6359** | 0.5960 |
| LUAD | **0.7096** | 0.6571 |
| UCEC | **0.6892** | 0.6675 |

Table S7. Hyperparameter of loss function weight utilized in TMO-Net model

| loss function | weight |
| --- | --- |
| $\mathcal{L}_{self}$ | 1.0 |
| $\mathcal{L}_{cross}$ | 0.1 |
| $\mathcal{L}_{con}$ | 0.1 |
| $\mathcal{L}_{dis}$ | 0.01 |

Table S8. Comparison of the effects of distinct loss ablations in cancer subtype classification task

| Cross loss | Dis. loss | Contrastive loss | ACC | Precision | Recall | F1-Score |
| --- | --- | --- | --- | --- | --- | --- |
| √ | √ | √ | **0.9445** | **0.8927** | **0.8817** | **0.8819** |
|  | √ | √ | 0.9240 | 0.8636 | 0.8484 | 0.8435 |
| √ |  | √ | 0.8829 | 0.8381 | 0.8070 | 0.8027 |
|  |  | √ | 0.8998 | 0.8284 | 0.8149 | 0.8035 |
|  | √ |  | 0.8245 | 0.7435 | 0.7172 | 0.7101 |
| √ | √ |  | 0.8005 | 0.7236 | 0.7119 | 0.7014 |
| √ |  |  | 0.7583 | 0.6260 | 0.6288 | 0.6087 |
|  |  |  | 0.7281 | 0.6410 | 0.5985 | 0.5961 |
