# Supplementary material for: TMO-Net: an explainable pretrained multi-omics model for multi-task learning in oncology
Source: Genome Biol. 2024 Jun 6;25:149. doi: 10.1186/s13059-024-03293-9 (PMC11157742; doi:10.1186/s13059-024-03293-9)
Supplement: Supplementary file 3 — Additional file 3: Review history. [file 13059_2024_3293_MOESM3_ESM.docx]

Review History

**First round of review**

**Reviewer 1**

**Were you able to assess all statistics in the manuscript, including the appropriateness of statistical tests used?**

Yes: I can assess the statistics.

**Were you able to directly test the methods?**

No.

**Comments to author:**

In this manuscript Wang et al. develop an interpretable deep learning model for integrating multimodal cancer omics datasets. In particular, the model can learn interactions between data modalities, infer joint representations, and even infer missing data. To demonstrate the use of this model, the authors trained the model with TCGA data and applied it to various scenarios, including predicting cancer types, breast cancer clinical subtypes, cancer metastatic status, response to drugs, and patient prognosis. The authors compared the performance of the model with other models/methods and show this model constantly outperforms others.

Interpretable deep learning models are a promising development in computational oncology. Such models have the potential to transform omics data analysis and even clinical development. Previously these models have been applied to individual omics data, but their integration has been increasingly appreciated for further improving their performance. In this regard, this study represents a significant advance and could be an important tool for mining cancer datasets.

Here below are my major comments:

1. Deep learning models usually require a large training dataset. TMO-Net is a pre-trained model, largely using the TCGA data. In its first application to predict cancer types (Fig 2), how did the authors split the TCGA data into training, testing, and validation? And what types of omics data were used when using the multi-omics as input? The authors compared multi-omics as input with gene expression as input. Among the multi-omics, which data type was the most informative and which were the least?

2. Fig 3 needs clarification. In Fig 3a, what does each row represent? And how did they choose the genes shown in the figure as opposed to other genes? Fig 3b could be confusing and easily misunderstood. If the purpose is to compare the correlation coefficients between those from TMO-Net and from "imputed", a boxplot should be much more straightforward. In addition, it is unclear how expression is imputed by the other method.

3. The authors tuned the model for various purposes. One way for such tuning is choosing different input data. For instance, for breast cancer subtype prediction (Fig 4), they used expression and mutation as input for model training. For metastasis status prediction (Fig 5), they used methylation and expression. For drug response (Fig 6), they used expression and mutation. Is there a rationale for choosing which types of data to use? Clarifying the rationale, if yes, would be helpful for understanding the pros and cons of using a particular type of data.

4. A highlight of the TMO-Net model is its interpretability. For instance, the authors identified genes that are important for breast cancer subtyping (Fig 4C) and pathways that are important for predicting responses to drugs (Fig 5C). Molecular subtypes of breast cancer are well established based on protein levels of estrogen receptor, HER2, etc. Interestingly, the authors' results show these markers not necessarily have a high important score. For instance, estrogen receptor did not make to the top list for luminal A. Can they shed some light on this? Similarly, the drugs Cetuximab and Erlotinib are EGFR inhibitors. Surprisingly no EGFR related pathways were on the top list (Fig 6c). This does not align with the mode of action of these drugs. It would be useful to discuss further on this observation.

5. Finally, in Fig 7, the authors stratified patients into distinct prognostic groups in 12 cancer types. Since TMO-Net was pretrained with 32 cancer types, this raises a question why the authors limited their analysis to only 12 cancer types.

**Reviewer 2**

**Were you able to assess all statistics in the manuscript, including the appropriateness of statistical tests used?**

Yes: Proper designed.

**Were you able to directly test the methods?**

Yes.

**Comments to author:**

Wang et al. presented a modeling study aimed at constructing a pre-trained multi-omics tumor network to capture interactions among diverse omics datasets, with subsequent application in various downstream tasks related to tumors. While the "pre-training and fine-tuning" framework is commonly employed in the training of large language or vision models, its application to biomedical datasets remains relatively uncommon, which was severely hidden by the absence of paired datasets and the complex data structure of biological datasets. This research addresses this gap in the context of cancer data and offers a timely contribution by proposing a potential workflow to improve the learning process for in-house small cohort and multi-omics cancer datasets. Moreover, it will further benefit the usage of the currently available tumor multi-omics datasets, such as TCGA, ICGC, and others. Overall, this paper is well-written and effectively organized.

This work has general significance to related research fields, however, I think the paper can be published after the following necessary revisions:

1. This paper evaluates the performance of TMO-Net models trained either with pre-training or from scratch, emphasizing the fundamental role of model pretraining in performance enhancement. Nevertheless, it is crucial to inquire about the impact of varying pretraining data scales on the final model performance.

2. Furthermore, the TCGA dataset is employed in a pan-cancer fashion during model pretraining. For specific tasks, such as breast cancer subtype prediction, it is essential to investigate whether the choice of cancer datasets for pretraining can influence the final performance. This consideration is vital in establishing the significance of model pretraining.

**Reviewer 3**

**Were you able to assess all statistics in the manuscript, including the appropriateness of statistical tests used?**

Yes: some statistics are missing such as imputed gene expression correlation, sample sizes (esp. for AUC).

**Were you able to directly test the methods?**

No.

**Comments to author:**

In this manuscript, the authors developed a deep learning pretrained model, TMO-Net to learn latent representations within and across multiple omics in cancers and use them to improve downstream predictions such as survival rates, drug responses. The TMO-Net model was based on joint variational autoencoders plus a later data fusion module to infer missing modalities. To this end, their data fusion step aligns latent spaces across different omics. For applications, they mainly trained the TMO-Net model using pan-cancer TCGA multiomics data (N=~8k) like gene expression, CNVs, mutations and methylation.

Overall, the TMO-Net model provides a valuable tool to learn meaningful multiomic latent features for cancer study, especially for survival analyses and drug responses as demonstrated in the paper. However, I have some major comments about the work, especially about the rigor and clarification.

* Many machine learning details for training TMO-Net are missing. Such insufficient description makes it challenging to evaluate the rigor of the model. These include like metadata for training, sample sizes, how to select features of each omics, addressing overfittings, cross-validation for downstream tasks (e.g., classification).

* The TMO-Net uses four losses. The authors need to justify the necessity of each loss (e.g., for different tasks) and guide how to tune lambda values.

* Similarly, are all omics needed to train a good TMO-Net? Actually, it is unclear that training was performed on the samples with all omics. If so, the statistical power can be a concern, especially using pan-cancer data.

* The data fusion to infer incomplete omics is not novel. Some recent papers like DeepGAMI (PMID: 37904203) can do cross-modality imputations on latent spaces. Plus, in Fig 3., the imputed gene expression values don't have high correlations, although significant p values. Can authors at least check if cancer-type maker genes are well imputed?

* The integrated gradient (IG) has been used to prioritize multiomic features such as Varmole (PMID: 33031552). The authors seem neglect those works and It is unclear how the authors implemented IG and how they used the IG values of each sample to prioritize other omic features besides genes.

* The manuscript may benefit further proofreading. For instance, Eq (14) is unclear such as the 2nd term for discriminator. Ref [16] is missing (only title).

* Ideally, the TMO-Net should be independently validated by other cohorts like ICGC.

**Authors Response**

**Point-by-point responses to the reviewers’ comments:**

**Reviewer #1:**

**Reviewer #1:** In this manuscript Wang et al. develop an interpretable deep learning model for integrating multimodal cancer omics datasets. In particular, the model can learn interactions between data modalities, infer joint representations, and even infer missing data. To demonstrate the use of this model, the authors trained the model with TCGA data and applied it to various scenarios, including predicting cancer types, breast cancer clinical subtypes, cancer metastatic status, response to drugs, and patient prognosis. The authors compared the performance of the model with other models/methods and show this model constantly outperforms others.

Interpretable deep learning models are a promising development in computational oncology. Such models have the potential to transform omics data analysis and even clinical development. Previously these models have been applied to individual omics data, but their integration has been increasingly appreciated for further improving their performance. In this regard, this study represents a significant advance and could be an important tool for mining cancer datasets.

***Response:*** *We appreciate Reviewer #1’s acknowledgment of our manuscript's contribution. By developing a multi-omics pre-training deep-learning model, we aimed to broaden our comprehension of tumorigenesis by utilizing the associations among various omics features. This approach not only enhances our understanding of cancer progression but also promises further cancer research, particularly in studies with small cohort sizes.*

**Here below are my major comments:**

**Comment-1:** Deep learning models usually require a large training dataset. TMO-Net is a pretrained model, largely using the TCGA data. In its first application to predict cancer types (Fig 2), how did the authors split the TCGA data into training, testing, and validation? And what types of omics data were used when using the multi-omics as input? The authors compared multi-omics as input with gene expression as input. Among the multi-omics, which data type was the most informative and which were the least?

***Response:*** *We thank Reviewer #1 for raising concerns about the application of the TCGA pancaner dataset during the training and testing phases of our model, and we apologize for the unclear description in the original manuscript. In the tumor subtype classification task, we utilized all multi-omics datasets for model pre-training, and the learned embeddings of all multi-omics datasets were obtained and used an XGBoost model to class cancer subtypes. In this way, we can assess the performance of different methods in sample representation learning. In model pretraining, we utilized four omics, including expression profiles, DNA methylation profiles, gene mutation data, and copy number variation (CNV) data. We have incorporated this clarification into the “Data processing” section of the Methods.*

*We agree with Reviewer #1 on the significance of discerning the contributions of different omics to model prediction. Following the official tutorial of the “Captum” interpretation method (https://captum.ai/tutorials/Multimodal_VQA_Interpret), we accessed the impact of different input omics by aggregating the attribution scores of individual features towards the tumor classification task. Specifically, we assessed the importance of various omics to the tumor subtypespecific SoftMax layer neurons, respectively. Our finding reveals that the “gene expression” and “DNA methylation” modalities* *are more important to the prediction result, whereas the “gene mutation” and “copy number variation” modalities have less impact (Table R1). This analysis has been incorporated into the revised manuscript to accurately reflect the contributions of different modalities in our study.*

Table R1. Contributions of different modalities in the pan-cancer classification task

| Cancer Type | gene expression | DNA methylation | gene mutation | copy number variation (CNV) |
| --- | --- | --- | --- | --- |
| ACC | 4.184484703 | 5.517831275 | 0.066589384 | 0.210567966 |
| BLCA | 4.437674079 | 5.232496711 | 0.334054839 | 0.135867182 |
| CESC | 4.35800042 | 5.216562296 | 0.058221816 | 0.16528564 |
| CHOL | 4.427356239 | 5.13624715 | 0.067397213 | 0.078667939 |
| COAD | 4.401682124 | 5.131295069 | 0.052219773 | 0.124388125 |
| DLBC | 4.239868785 | 5.470517208 | 0.037125865 | 0.088048783 |
| ESCA | 4.609513871 | 5.081509663 | 0.01394069 | 0.158046025 |
| GBM | 4.096903024 | 4.589240368 | 0.033438703 | 0.19819148 |
| HNSC | 4.315672131 | 5.10903255 | 0.031113458 | 0.214965488 |
| KICH | 4.214348759 | 5.143230883 | 0.028312496 | 0.126080668 |
| KIRC | 4.531865731 | 5.116248582 | 0.021413437 | 0.213448106 |
| KIRP | 4.237678491 | 4.872835933 | 0.044965904 | 0.153029751 |
| LGG | 4.389836642 | 4.982489999 | 0.060528383 | 0.185041886 |
| LIHC | 4.30289782 | 4.970509655 | 0.041678019 | 0.169126942 |
| LUAD | 4.304432749 | 5.224168475 | 0.02495416 | 0.106891649 |
| LUSC | 3.939880564 | 5.439625999 | 0.018476779 | 0.172905915 |
| MESO | 4.291522628 | 5.272573557 | 0.017802219 | 0.058097735 |
| OV | 4.532025793 | 4.784307117 | 0.030644862 | 0.083449735 |
| PAAD | 4.089159245 | 4.871647006 | 0.064007792 | 0.146897458 |
| PCPG | 4.129293588 | 4.65820761 | 0.047535296 | 0.170220906 |
| PRAD | 4.594854049 | 5.228774306 | 0.084154532 | 0.116222997 |
| READ | 4.074443706 | 5.240295703 | 0.093657133 | 0.06777761 |
| SARC | 4.158341648 | 4.922763834 | 0.051513842 | 0.168863694 |
| SKCM | 4.369942092 | 4.863415831 | 0.168784381 | 0.185336623 |
| STAD | 4.319955737 | 5.257354216 | 0.022483888 | 0.089889297 |
| TGCT | 4.112766208 | 4.666435804 | 0.063157297 | 0.075808525 |
| THCA | 3.93166193 | 4.772822791 | 0.050800902 | 0.195134507 |
| THYM | 4.183466653 | 4.680707197 | 0.048143491 | 0.226514558 |
| UCEC | 3.734388844 | 4.64334665 | 0.340907368 | 0.096524855 |
| UCS | 4.115386318 | 4.588737961 | 0.051243415 | 0.13184302 |
| UVM | 4.192120043 | 5.068121137 | 0.173226445 | 0.11884854 |
| BRCA | 4.130648826 | 5.205548652 | 0.062417908 | 0.144165628 |

**Comment-2:** Fig 3 needs clarification. In Fig 3a, what does each row represent? And how did they choose the genes shown in the figure as opposed to other genes? Fig 3b could be confusing and easily misunderstood. If the purpose is to compare the correlation coefficients between those from TMO-Net and from "imputed", a boxplot should be much more straightforward. In addition, it is unclear how expression is imputed by the other method.

***Response:*** *We thank Reviewer #1 for highlighting the insufficient description in Figure 3. In Figure 3, we aimed to present the capability of our model in imputing missing modalities. Specifically, in Fig 3a, each row corresponds to different test tumor samples, with the color bar representing either the raw or the inferred expression values of the randomly selected genes. In Fig 3b, we utilized a scatter plot to indicate the correlation coefficients between raw and inferred gene expression across tumor samples using different methods, accompanied by a density plot that highlights the superior performance of the TMO-Net model in gene expression inference when compared to a prediction-only neural network.*

*To better validate the efficacy of our model in cross-modality learning, we revised Figure 3 to incorporate two prediction tasks. The first one is to leverage other modalities to predict gene expressions, and the other one is to predict gene mutations. In the gene expression prediction task, we utilized Pearson’s correlation coefficients and coefficient of determination (R^2^) to quantify the capability of modality inference (Fig R1a-c). In the task of gene mutation prediction, we applied the Area under the curve (AUC) score to evaluate the performance of this model in predicting tumor-specific gene mutations for other modalities (Fig R1d, e). Additionally, we used a dualautoencoder model as a baseline for comparison, as described in Tang et al. [1]. The results demonstrate that our modal achieves higher modality inference performance, suggesting an enhanced comprehension of modality interactions.*

Fig R1. Performance comparison of TMO-Net model in gene expression and mutation imputation tasks. a and b, Comparison of the averaged coefficient of determination (*R^2^*) (a) and Pearson’s correlation coefficients (b) of raw and reconstructed gene expression values from other modalities. (c) Heatmap plot of the raw and reconstructed gene expressions in selected genes across cancer subtypes. (d) Comparison of the averaged AUC score of predicted gene mutations in the top 50 most mutated genes. (e) Receiver Operating Characteristic (ROC) plots of the prediction result of typical cancer mutations. 5-fold crossvalidation results were presented in (a, b, and d), with mean ± SD.

**Comment-3:** The authors tuned the model for various purposes. One way for such tuning is choosing different input data. For instance, for breast cancer subtype prediction (Fig 4), they used expression and mutation as input for model training. For metastasis status prediction (Fig 5), they used methylation and expression. For drug response (Fig 6), they used expression and mutation. Is there a rationale for choosing which types of data to use? Clarifying the rationale, if yes, would be helpful for understanding the pros and cons of using a particular type of data.

***Response:*** *We thank Reviewer #1 for emphasizing the need for clarity regarding the use of datasets in various downstream tasks. In this study, we aimed to construct a deep-learning model that can learn multi-omics cancer datasets via the model pre-training phase, and then adapt this model to various downstream tasks utilizing new multi-omics datasets through model fine-tuning. A critical feature of this model is its capability in incomplete-omics data learning, which will enhance the utilization of new tumor datasets with limited modalities and cohort size.*

*To assess our model’s efficacy, we conducted several downstream tasks, including breast cancer subtype classification, drug response prediction, and metastasis prediction. The criteria for selecting these multi-omics datasets include shared data modality usage and similar data preprocessing procedures. Additionally, these datasets have been utilized by other baseline multiomics deep learning models. For the downstream analysis, we did not select specific modalities but utilized all modalities from these datasets. This approach allows us to make direct comparisons with other baseline methods that utilize the same datasets. We apologize for any lack of clarity in our original manuscript regarding the application of multi-omics datasets in downstream tasks and will include this detailed explanation in the “Downstream task model fine-tuning” section of our revised manuscript.*

Comment 4: A highlight of the TMO-Net model is its interpretability. For instance, the authors identified genes that are important for breast cancer subtyping (Fig 4C) and pathways that are important for predicting responses to drugs (Fig 5C). Molecular subtypes of breast cancer are well established based on protein levels of estrogen receptor, HER2, etc. Interestingly, the authors' results show these markers not necessarily have a high important score. For instance, estrogen receptor did not make to the top list for luminal A. Can they shed some light on this? Similarly, the drugs Cetuximab and Erlotinib are EGFR inhibitors. Surprisingly no EGFR related pathways were on the top list (Fig 6c). This does not align with the mode of action of these drugs. It would be useful to discuss further on this observation.

***Response:*** *We thank Reviewer #1 for pointing out the need for a more detailed explanation of our data interpretation analysis. In this study, we employed the Integrated Gradients (IG) method to assess the attribution values of input modalities. This approach is based on the premise that the contribution of an input feature to the model's prediction should correspond to the change in the model's output as the feature's value transitions from a baseline to its actual value. The IG values' magnitude signifies their impact on model predictions, while their sign indicates the input features' regulatory roles as either positive or negative.*

*In this study, we used the absolute IG value to indicate the importance of input features to avoid the elimination of positive and negative IG values in sample group analysis, such as in breast cancer subtype prediction, aligning with the methodology of Janizek et al. [2]. Thus, the ranked gene features represent their importance but ignore their effects on the prediction outcomes, such as the classification probability of Luminal A and B subtypes. Specifically, we have identified the gene ESR1 that related to the Estrogen* *Receptor Signaling is highly ranked in ER- (Basel and HER2) tumor subtypes, highlighting the absent expression of this gene is also a critical feature in subtype classification. To validate that this method can reflect the true biology signal, we compared the raw IG values of gene ESR1 (related to ER+ subtypes) and gene ERBB2 (related to HER2 subtypes) in different breast cancer subtypes (Fig R2), the results indicated the positive gene IG values in specific cancer subtypes and accordant with its biology background.*

*In drug response prediction analysis, limited by the size of validating datasets, we have only 60 samples treated with Cetuximab, and 21 samples treated with Erlotinib. We then compared the correlation coefficients between the raw IG values of specific samples with their EGFR expression values. Correlation analyses revealed a significant correlation in cetuximab-treated samples, proposing the critical roles of EGFR expression level in Cetuximab drug responses (Fig. R3). However, we have not identified a high correlation in Erlotinib-treated samples (Fig. R3), which may be retrained by the limited sample size and potential drug resistance. We will highlight this limitation in the revised manuscript.*

*In summary, the interpretation analysis in our study can highlight the important molecular features in model prediction, and their further roles in disease diagnosis and treatment design require further discussions.*

Fig R2. Raw IG values of breast cancer subtype-specific gene expression features.

Fig R3. Correlation analyses of the *EGFR* expression values and their IG values in different *EGFR* inhibitors.

**Comment 5:** Finally, in Fig 7, the authors stratified patients into distinct prognostic groups in 12 cancer types. Since TMO-Net was pretrained with 32 cancer types, this raises a question why the authors limited their analysis to only 12 cancer types.

***Response:*** *We thank Reviewer#1 for raising the concerns about the tumor subtype selected in prognosis prediction, and apologize for the unclear description in the tumor subtype selection criterion. In our study, tumor subtypes with more than 50 uncensored samples were selected for the prognosis prediction task, as the limited cohort sizes and high ratio of censored follow-up data could affect the prognosis prediction.*

*Additionally, as suggested by Reviewer #3, we have conducted an extra experiment to further validate our model’s effectiveness in prognosis prediction (Table R5). This experiment utilized the independent CPTAC multi-omics dataset. The outcomes of this experiment confirmed the superior capability of the TMO-Net model in prognosis prediction, benefiting from the model pre-training.*

**Reviewer #2:**

**Reviewer #2:** Wang et al. presented a modeling study aimed at constructing a pre-trained multiomics tumor network to capture interactions among diverse omics datasets, with subsequent application in various downstream tasks related to tumors. While the "pre-training and finetuning" framework is commonly employed in the training of large language or vision models, its application to biomedical datasets remains relatively uncommon, which was severely hidden by the absence of paired datasets and the complex data structure of biological datasets. This research addresses this gap in the context of cancer data and offers a timely contribution by proposing a potential workflow to improve the learning process for in-house small cohort and multi-omics cancer datasets. Moreover, it will further benefit the usage of the currently available tumor multiomics datasets, such as TCGA, ICGC, and others. Overall, this paper is well-written and effectively organized.

This work has general significance to related research fields, however, I think the paper can be published after the following necessary revisions:

***Response:*** *We are grateful to Reviewer #2 for highlighting the contribution of our manuscript. In this study, we aimed to apply the "pre-training and fine-tuning" framework to fully utilize cancer multi-omics datasets for multiple downstream tasks with limited cohort size or incomplete data modalities. As suggested by Reviewer #2, we have conducted multiple experiments to interrogate the contributions of pre-training data scales and cancer subtypes in modal performance*.

**Comment 1:** This paper evaluates the performance of TMO-Net models trained either with pretraining or from scratch, emphasizing the fundamental role of model pretraining in performance enhancement. Nevertheless, it is crucial to inquire about the impact of varying pretraining data scales on the final model performance.

***Response:*** *We thank Reviewer#2 for emphasizing the significance of the pre-training dataset scale in our research. In this study, we have assessed the performance difference between pre-trained and trained from scratch TMO-Net models, the results demonstrated the performance improvement through pan-caner multi-omics data pre-training. As suggested by Reviewer #2, we conducted a series of experiments with diverse scales of pre-training datasets, ranging from 10% to 100% of multi-omics training data, subset by individual tumor subtypes. These experiments were then evaluated using a validation dataset. The performance of pre-trained models across varying scales of training data in tumor subtype classification tasks underscores* *the alignment between training data scale and model performance (Fig R4, Table R2), further validating the importance of model pre-training in our model.*

Fig R4. Comparison of the performance of cancer subtype classification task with different scales of pretraining multi-omics dataset.

Table R2. Contributions of pre-training data scales in the pan-cancer classification task

| Data size | ACC | Precision | Recall | F1 score |
| --- | --- | --- | --- | --- |
| 654 (10%) | 0.3975 | 0.2239 | 0.2353 | 0.1989 |
| 1308 (20%) | 0.7097 | 0.5582 | 0.5446 | 0.5242 |
| 1962 (30%) | 0.7383 | 0.6776 | 0.5997 | 0.5942 |
| 2616 (40%) | 0.8009 | 0.7219 | 0.6842 | 0.6786 |
| 3270 (50%) | 0.8286 | 0.7550 | 0.7097 | 0.7005 |
| 3924 (60%) | 0.8226 | 0.7643 | 0.7627 | 0.7567 |
| 4577 (70%) | 0.8305 | 0.7683 | 0.7581 | 0.7546 |
| 5231 (80%) | 0.8905 | 0.8487 | 0.8264 | 0.8310 |
| 5885 (90%) | 0.8960 | 0.8316 | 0.8214 | 0.8138 |
| 6539 (100%) | **0.9445** | **0.8927** | **0.8817** | **0.8819** |

**Comment 2:** Furthermore, the TCGA dataset is employed in a pan-cancer fashion during model pretraining. For specific tasks, such as breast cancer subtype prediction, it is essential to investigate whether the choice of cancer datasets for pretraining can influence the final performance. This consideration is vital in establishing the significance of model pretraining.

***Response:*** *We thank Reviewer#2 for highlighting the potential effects of different pre-training datasets in model performance. To understand the effects of different pre-training datasets on model performance. We conducted experiments to pre-train TMO-Net models with and without TCGA-BRCA multi-omics datasets, to assess the impact of similar pre-training datasets in the breast cancer subtype classification task. The classification performance of different models was then evaluated with the PAM50 classification task and fine-tuned with the independent METABRIC datasets. Our results of the breast cancer classification task with the testing METABRIC dataset revealed that the TMO-Net model pre-trained with the TCGA-BRCA dataset can enhance the performance of downstream tasks (Table R3), suggesting a similar and highquality pre-training dataset can benefit downstream oncology tasks.*

Table R3. Comparison of different pre-training datasets in the PAM50 subtype classification task

| Models | ACC | Precision | Recall | F1-Score |
| --- | --- | --- | --- | --- |
| TMO-Net (without TCGA-BRCA) | 0.8481 | 0.8526 | 0.8481 | 0.8590 |
| TMO-Net (with TCGA-BRCA) | **0.8860** | **0.8901** | **0.8860** | **0.8901** |

**Reviewer #3:**

**Reviewer #3:** In this manuscript, the authors developed a deep learning pretrained model, TMONet to learn latent representations within and across multiple omics in cancers and use them to improve downstream predictions such as survival rates, drug responses. The TMO-Net model was based on joint variational autoencoders plus a later data fusion module to infer missing modalities. To this end, their data fusion step aligns latent spaces across different omics. For applications, they mainly trained the TMO-Net model using pan-cancer TCGA multiomics data (N=~8k) like gene expression, CNVs, mutations and methylation.

Overall, the TMO-Net model provides a valuable tool to learn meaningful multiomic latent features for cancer study, especially for survival analyses and drug responses as demonstrated in the paper. However, I have some major comments about the work, especially about the rigor and clarification.

***Response:*** *We appreciate Reviewer #3 for highlighting the significance of our study in the context of multi-omics latent learning. By integrating the potential feature associations across different data modalities via model pre-training, our objective was to enhance the data learning of small cohort datasets with incomplete multi-omics datasets.*

**Comment 1:** Many machine learning details for training TMO-Net are missing. Such insufficient description makes it challenging to evaluate the rigor of the model. These include like metadata for training, sample sizes, how to select features of each omics, addressing overfittings, crossvalidation for downstream tasks (e.g., classification).

***Response:*** *We thank Reviewer #3 for highlighting the drawbacks and limitations in the method description, and we apologize for any ambiguity present in the original manuscript. In response, we have extensively revised the method section in the revised manuscript, providing clearer explanations. We have updated the sample size information and feature selection criterion in the "Data preprocessing" section of the methods. Additionally, we introduced detailed processes for model pre-training and fine-tuning in the "TMO-Net pre-training process" and "Downstream task model fine-tuning process" sections of the methods.*

**Comment 2:** The TMO-Net uses four losses. The authors need to justify the necessity of each loss (e.g., for different tasks) and guide how to tune lambda values.

***Response:*** *We are grateful to Reviewer #3 for highlighting the concern regarding the absence of ablation tests in our study. To understand the effects of different losses in this study, we conducted a series of loss* *ablation experiments to assess the impact of different loss designs on the pan-cancer subtype classification task (Table R4). The results of these experiments confirmed that all losses incorporated in the TMO-Net model contribute to enhancing model performance in pan-cancer subtype learning. Specifically, the contrastive loss emerged as the most critical loss in the pancaner subtype learning, followed by the adversarial loss of discriminators and cross-model VAE loss. Furthermore, the optimal combination of hyperparameters and loss function weights in the TMO-Net model was determined through a grid search, and evaluated with a 5-fold crossvalidation, and we have listed the hyperparameters used in this study in the additional file of the revised manuscript.*

Table R4. Comparison of the effects of distinct loss ablations in cancer subtype classification task

| Cross loss | Dsc. loss | Contrastive loss | ACC | Precision | Recall | F1-Score |
| --- | --- | --- | --- | --- | --- | --- |
| √ | √ | √ | 0.9445 | 0.8927 | 0.8817 | 0.8819 |
|  | √ | √ | 0.9240 | 0.8636 | 0.8484 | 0.8435 |
| √ |  | √ | 0.8829 | 0.8381 | 0.8070 | 0.8027 |
|  |  | √ | 0.8998 | 0.8284 | 0.8149 | 0.8035 |
|  | √ |  | 0.8245 | 0.7435 | 0.7172 | 0.7101 |
| √ | √ |  | 0.8005 | 0.7236 | 0.7119 | 0.7014 |
| √ |  |  | 0.7583 | 0.6260 | 0.6288 | 0.6087 |
|  |  |  | 0.7281 | 0.6410 | 0.5985 | 0.5961 |

Comment 3: Similarly, are all omics needed to train a good TMO-Net? Actually, it is unclear that training was performed on the samples with all omics. If so, the statistical power can be a concern, especially using pan-cancer data.

***Response:*** *We are grateful to Reviewer #3 for bringing up concerns regarding the effects of selected modalities and the scales of pre-training datasets in model pre-training, which is a fundamental aspect of multi-modal learning. As emphasized by all three reviewers, we have implemented methods and conducted experiments to assess the contributions of both pre-training data scales and cancer-specific datasets in model training. Our results indicated the significant contribution and priority of gene expression and DNA methylation modalities in multi-omics pancancer subtype learning (Table R1). Through comparisons of pre-training data scales and specific cancer pre-training data, we elucidated that larger and more relevant pre-training datasets positively influence model performance (Fig R4, Table R2, 3). In line with our results,* *a theoretical study has demonstrated that multi-modal learning can more accurately estimate the sample latent representations [3].*

*However, due to the limited availability of large paired multi-omics cancer datasets, our evaluation of the associations between pre-training datasets and the subsequent statistical power of trained models remains incomplete. Moreover, determining the essential data scales or modalities necessary for multimodal model training can be subjective and related to the model task and modal frameworks. Additionally, given the expanding multi-omics datasets in cancer research, we anticipate that these datasets can be further leveraged to uncover regulatory mechanisms in tumorigenesis and development, aligning with advancements in large language or vision multimodal learning models.*

**Comment 4:** The data fusion to infer incomplete omics is not novel. Some recent papers like DeepGAMI (PMID: 37904203) can do cross-modality imputations on latent spaces. Plus, in Fig 3., the imputed gene expression values don't have high correlations, although significant p values.

Can authors at least check if cancer-type maker genes are well imputed?

***Response:*** *We agree with Reviewer #3 that the cross-modality imputation is not a novel concept in multi-omics biological data learning. Indeed, numerous studies have leveraged cross-modality learning to explore associations across different modality features [4-7]. We will incorporate these references into the introduction section of the revised manuscript. In our study, our objective was to apply the principle of cross-modality learning to address the challenge of incomplete multiomics representation learning of cancer samples. By learning shared latent embeddings of multiomics tumor data and enabling inference across modalities, our study proposed a versatile multiomics learning framework adaptable to various downstream tasks in cancer research.*

*As also commented by Reviewer #2, we have reorganized Figure 3 to better characterize the performance of our model in cross-modal inference. Additionally, we introduced a new baseline dual-autoencoder model for reference. Both the gene expression and mutation inference tasks have validated our model's superiority in cross-modal learning (Fig R1). In line with Reviewer #3's suggestion, we compared our model's performance in predicting cancer-specific gene mutation (Fig R1d, e), and the result revealed our model can better infer gene mutations from other modalities.*

**Comment 5:** The integrated gradient (IG) has been used to prioritize multiomic features such as Varmole (PMID: 33031552). The authors seem neglect those works and It is unclear how the authors implemented IG and how they used the IG values of each sample to prioritize other omic features besides genes.

***Response:*** *We thank Reviewer #3 for emphasizing the importance of interactive interpretation analysis between cross-modality features, which can be further applied to explore molecular regulatory mechanisms, such as the expression Quantitative Trait Loci (eQTL) analysis across diverse disease phenotypes. As recommended by Reviewer #3, the Varmole method has integrated a specialized layer to represent proxy gene features to elucidate associations between gene variants and gene expressions. However, we acknowledge that despite the potential associations across different modality features accessible through the cross-modal inference task in our model, the lack of biological knowledge and specific network design may impede further exploration of gene regulatory mechanisms in tumor research. We concur with Reviewer #3 on the significance of this aspect for advancing multi-omics cancer research. Although it is beyond the scope of our current model, addressing this important task will be a key objective in our future work.*

*In response to comments from Reviewers #1 and #3, we have revised the description of data interpretation methods in the “Model interpretation analysis” section of the methods. In summary, we utilized the absolute IG values of input molecular features associated with task-specific neurons to assess the contributions of specific features or individual modalities to model outcomes.*

**Comment 6:** The manuscript may benefit further proofreading. For instance, Eq (14) is unclear such as the 2nd term for discriminator. Ref [16] is missing (only title).

***Response:*** *We thank Reviewer #3 for raising the concerns of the method description in our initial manuscript. We have intensively revised our manuscript, addressing all equations and references in the revised manuscript to ensure accuracy and clarity.*

**Comment 7:** Ideally, the TMO-Net should be independently validated by other cohorts like ICGC.

***Response:*** *We appreciate Reviewer #3 for highlighting the importance of conducting an additional independent validation in our study. To validate the efficacy of data pre-training in the context of multi-omics cancer dataset learning, we leveraged independent datasets from the Clinical Proteomic Tumor Analysis Consortium (CPTAC) project for model pre-training, subsequently evaluating performance using a survival prediction task. Specifically, we obtained the multi-omics cancer datasets and associated clinical profiles from the LinkedOmics platform (http://www.linkedomics.org). Following data preprocessing and feature selection for model training and testing, we compared the Concordance Index (C-index) of both the pre-trained TMONet model and a TMO-Net model trained from scratch in different cancer subtypes (Table R5). Our findings validate that our model effectively utilizes pre-training datasets, thereby enhancing the performance of prognosis prediction in independent multi-omics cancer datasets. These results confirm that employing a data pre-training strategy can significantly enhance the performance of multimodal deep-learning models in cancer research. Moreover, such an approach can prove especially beneficial for tumor research with limited cohort sizes or incomplete data modalities.*

Table R5. Validation of the TMO-Net model using independent CPTAC datasets

| Cancer subtype | TMO-Net  (pre-trained) | TMO-Net  (trained from scratch) |
| --- | --- | --- |
| COAD | 0.7973 | 0.6060 |
| GBM | 0.6359 | 0.5960 |
| LUAD | 0.7096 | 0.6571 |
| UCEC | 0.6892 | 0.6675 |

***Reference***

1. *Tang X, Zhang J, He Y, Zhang X, Lin Z, Partarrieu S, Hanna EB, Ren Z, Shen H, Yang Y, et al:* ***Explainable multi-task learning for multi-modality biological data analysis.*** *Nat Commun 2023,* ***14:****2546.*
2. *Janizek JD, Spiro A, Celik S, Blue BW, Russell JC, Lee TI, Kaeberlin M, Lee SI:* ***PAUSE:***

***principled feature attribution for unsupervised gene expression analysis.*** *Genome Biol 2023,* ***24:****81.*

3. *Huang Y, Du C, Xue Z, Chen X, Zhao H, Huang L:* ***What makes multi-modal learning better than single (provably).*** *Advances in Neural Information Processing Systems 2021,* ***34:****10944-10956.*
4. *Alatkar SA, Wang D:* ***CMOT: Cross-Modality Optimal Transport for multimodal inference.*** *Genome Biol 2023,* ***24:****163.*
5. *Wu KE, Yost KE, Chang HY, Zou J:* ***BABEL enables cross-modality translation between multiomic profiles at single-cell resolution.*** *Proc Natl Acad Sci U S A 2021,* ***118****.*
6. *He Z, Hu S, Chen Y, An S, Zhou J, Liu R, Shi J, Wang J, Dong G, Shi J:* ***Mosaic integration and knowledge transfer of single-cell multimodal data with MIDAS.*** *Nature Biotechnology 2024****:****1-12.*
7. *Chandrashekar PB, Alatkar S, Wang J, Hoffman GE, He C, Jin T, Khullar S, Bendl J, Fullard JF, Roussos P, Wang D:* ***DeepGAMI: deep biologically guided auxiliary learning for multimodal integration and imputation to improve genotype-phenotype prediction.*** *Genome Med 2023,* ***15:****88.*

**Second round of review**

**Reviewer 1**

The authors have addressed my comments.

**Reviewer 2**

My comments are properly addressed.
